# Increased oxidative stress contributes to impaired peripheral CD56^dim^CD57^+^ NK cells from patients with systemic lupus erythematosus

**DOI:** 10.1186/s13075-022-02731-y

**Published:** 2022-02-16

**Authors:** Zhimin Lu, Yao Tian, Ziran Bai, Jiaqing Liu, Yan Zhang, Jingjing Qi, Minli Jin, Jie Zhu, Xia Li

**Affiliations:** 1 Department of Immunology, College of Basic Medical Science, Dalian Medical University, Dalian, People’s Republic of China; 2 Department of Rheumatology, Affiliated Hospital of Nantong University, Nantong, People’s Republic of China; 3 Flow Cytometry Center, The Second Hospital of Dalian Medical University, Dalian, People’s Republic of China; 4 Department of Rheumatology, The Second Hospital of Dalian Medical University, Dalian, People’s Republic of China

**Keywords:** Systemic lupus erythematosus, Natural killer cell, Subset, Apoptosis, Reactive oxygen species

## Abstract

**Background:**

Systemic lupus erythematosus (SLE) is characterized by loss of immune tolerance and imbalance of immune cell subsets. Natural killer (NK) cells contribute to regulate both the innate and adaptive immune response. In this study, we aimed to detect alterations of peripheral NK cells and explore intrinsic mechanisms involving in NK cell abnormality in SLE.

**Methods:**

Blood samples from healthy controls (HCs) and patients with SLE and rheumatoid arthritis (RA) were collected. The NK count, NK subsets (CD56^bright^, CD56^dim^CD57^−^, and CD56^dim^CD57^+^), phenotypes, and apoptosis were evaluated with flow cytometer. Mitochondrial reactive oxygen species (mtROS) and total ROS levels were detected with MitoSOX Red and DCFH-DA staining respectively. Published data (GSE63829 and GSE23695) from Gene Expression Omnibus (GEO) was analyzed by Gene Set Enrichment Analysis (GSEA).

**Results:**

Total peripheral NK count was down-regulated in untreated SLE patients in comparison to that in untreated RA patients and HCs. SLE patients exhibited a selective reduction in peripheral CD56^dim^CD57^+^ NK cell proportion, which was negatively associated with disease activity and positively correlated with levels of complement(C)3 and C4. Compared with HCs, peripheral CD56^dim^CD57^+^ NK cells from SLE patients exhibited altered phenotypes, increased endogenous apoptosis and higher levels of mtROS and ROS. In addition, when treated with hydrogen peroxide (H_2_O_2_), peripheral CD56^dim^CD57^+^ NK cell subset was more prone to undergo apoptosis than CD56^dim^CD57^−^ NK cells. Furthermore, this NK cell subset from SLE patients exhibited impaired cytotoxicity in response to activated CD4^+^ T cells in vitro.

**Conclusion:**

Our study demonstrated a selective loss of mature CD56^dim^CD57^+^ NK cell subset in SLE patients, which may caused by preferential apoptosis of this subset under increased oxidative stress in SLE. The attenuated in vitro cytotoxicity of CD56^dim^CD57^+^ NK cells may contribute to the impaired ability of eliminating pathogenic CD4^+^ T cells in SLE.

**Supplementary Information:**

The online version contains supplementary material available at 10.1186/s13075-022-02731-y.

## Background

Systemic lupus erythematosus (SLE) is a multi-organ autoimmune disease driven by aberrant activation of autoreactive T and B cells and the production of autoantibodies [1]. Patients with SLE are known to exhibit diminished antiviral immunity and heightened risk for the development of severe opportunistic infections, which represents an important cause of mortality [2].

NK cells are innate lymphoid cells which mediate resistance against tumor cells and microbial pathogens, especially the virus, and also contribute to the regulation of adaptive immune response [3, 4]. NK cells exert cytotoxicity through expresssion of CD107a, production of lytic granules (e.g. perforin and granzyme B) and engagement of CD16. They also regulate the immune response by cytokine production, such as IFN-γ and IL-10. In humans, NK cells have long been classified into two well-characterized subsets, known as CD56^dim^ NK cells and CD56^bright^ NK cells. The former, with stronger cytotoxic activity, are more mature and predominant in the peripheral blood. The latter, with more cytokine production, are predominant in tissues [5]. Recent studies suggest that CD57 expression marks mature human NK cells and CD56^dim^CD57^+^ NK cell is a newly discovered important subgroup, which is characterized by enhanced lytic granule protein contents, including perforin and granzyme B [5–7]. Furthermore, transcriptome and protein expression data have displayed gradients in cell activities from CD56^bright^ NK cells to CD56^dim^CD57^−^ NK cells to CD56^dim^CD57^+^ NK cells [7].

NK cells have been shown to play a beneficial role in the regulation of pathogenic autoreactive T cell and B cell responses [3, 4]. NK cell depletion accelerates disease activity in lupus-prone mice, whereas NK cell transfer delays disease onset [8]. A transcriptome level study suggests that NK cell cytotoxicity transcript is negatively correlated with lupus disease activity [9]. Number reductions and phenotype alterations of NK cells in SLE patients have been described [10–12], but the results are not consistent. The discrepancies in the above studies may be explained by differences in treatment regimen and NK cell heterogeneity, as total NK cells rather than NK cell subsets were detected in most studies.

We conducted this study with the purpose of detecting the number and the subsets of NK cells in peripheral blood from SLE patients and their correlations with disease activity. Furthermore, we investigated their cytotoxic effect on activated CD4^+^ T cells, and explored the intrinsic mechanisms which may contribute to NK cell abnormality in SLE patients.

## Methods

### Study subjects

All the samples were obtained from individuals fulfilling the diagnosis of SLE or rheumatoid arthritis (RA) according to the American College of Rheumatology (ACR) criteria [13, 14] in Affiliated Hospital of Nantong University and The Second Hospital of Dalian Medical University (from 2017 to 2020). Patients complicated with tumor, infections or other autoimmune diseases were excluded. Healthy controls (HCs) were chosen from age and gender matched healthy volunteers. Our study was approved by the ethics committee of the Affiliated Hospital of Nantong University and Dalian Medical University.

### Baseline characteristics

Related test results of the subjects enrolled were collected through review of medical records, including: age, gender, duration, white blood cell (WBC), hemoglobin (Hb), platelet (PLT), erythrocyte sedimentation rate (ESR), C-reactive protein (CRP), complement C3 and C4, anti-double-stranded-DNA antibodies (anti-dsDNA antibodies), antinuclear antibodies (ANA), immunoglobulin G (IgG), immunoglobulin M (IgM), immunoglobulin A (IgA), and albumin (ALB). Disease activity was measured using the Systemic Lupus Erythematosus Disease Activity Index 2000 (SLEDAI) score for SLE patients [15]. Disease activity was measured using 28-joint disease activity score (DAS28) for RA patients. Lupus nephritis (LN) is defined as proteinuria (more than 0.5 g of protein per day or 3 + on dipstick) or the presence of cellular casts (either red blood cell, hemoglobin, granular, tubular or mixed) in urinary sediment or renal biopsy.

### Total NK cell analysis in treatment-naive patients

We conducted 156 treatment-naive SLE patients and 22 treatment-naive RA patients with no history of glucocorticoids or immunosuppressive drugs. 30 age and gender matched HCs were enrolled. Blood cells were incubated with a panel of monoclonal antibodies (anti-human-CD3, anti-human-CD56). Trucount tubes were used to determine the absolute number. The count and percentage of CD3^−^CD56^+^ NK cells were measured.

### NK subset analysis by flow cytometry

We conducted 37 untreated SLE patients and 30 HCs matched for age and gender. Blood samples were stained with fluorophores conjugated antibodies for phenotypic detecting. Peripheral blood mononuclear cells (PBMCs) were isolated using Ficoll density gradient centrifugation (400 g, 30 min) for cell stimulation. The NK cell subset gating strategies were depicted in Supplementary Fig. 1. All antibodies for staining were shown in Supplementary Table 1. Cell suspensions were surface-labeled with antibodies for 30 min. For intracellular staining, cell suspensions were firstly incubated with surface antibody, then fixed and permeabilized using the Transcription Factor Fix/Perm Buffer (eBioscience). For IFN-γ and IL-10 production assays, cells were stimulated with phorbol 12-myristate 13-acetate (PMA, 50 ng/ml) and ionomycin (1 µg/ml) for 4 h in the presence of Brefeldin A (10 µg/ml). All the cell samples were detected by BD FACSCanto™ II or ACEA NovoCyte and the acquired data were further analyzed with BD FACSDiva software or FlowJo software.

### Apoptosis detection

Apoptosis of NK cell subsets was detected by staining fresh PBMCs with annexin V (Invitrogen) and 7-amino-actinomycin D (7-AAD) (BD Biosciences) in 19 SLE patients and 10 HCs.

### Measurement of reactive oxygen species(ROS) levels

ROS levels were assessed by incubating cells with 10 µM 2',7'-Dichlorodihydrofluorescein diacetate (DCFH-DA) (AAT Bioquest) for 20 min at 37℃. Mitochondrial reactive oxygen species (mtROS) levels were assessed by incubating cells with 5 µM MitoSOX Red probe (invitrogen) for 15 min at 37℃. DCFH-DA and MitoSOX fluorescence intensities in NK cells were detected by flow cytometry as mean fluorescence intensity (MFI).

### Isolation of NK cell subsets

Total NK cells (> 92% purity) were negatively isolated by magnet associated cell sorting (MACS) (Biolegend). CD56^dim^CD57^+^ NK cells and CD56^dim^CD57^−^ NK cells (> 95% purity of each subset) were isolated from fresh PBMCs by fluorescence activated cell sorting (FACS) using a FACSAria II cell sorter (BD).

### Detection of CD107a expression

MACS-sorted NK cells were stimulated with PMA (50 ng/ml) and ionomycin (1 µg/ml) for 6 h in the presence of Brefeldin A (10 µg/ml) and PE-conjugated anti-CD107a in RPMI 1640 medium (10% FBS) at 37℃ in a 5% CO_2_ atmosphere. The expressions of CD107a on CD56^dim^CD57^+^ NK cells and CD56^dim^CD57^−^ NK cells were detected by Flow cytometry.

### Cytoxicity assay

Purified CD4^+^ T cells (> 95% purity) (Biolegend) were labeled with 1 µM carboxyfluorescein diacetate succinimidyl ester (CFSE) (Invitrogen) and rested or activated in RPMI 1640 medium (10% FBS) with human T-activator CD3/CD28 antibody (both 2 µg/ml, eBioscience) for 72 h. FACS-sorted NK cell subsets (CD56^dim^CD57^+^ NK cells and CD56^dim^CD57^−^ NK cells) were cocultured with these CD4^+^ T cells at a ratio of 1:1 for 15 h in RPMI 1640 medium (10% FBS) at 37℃ in a 5% CO_2_ atmosphere. FACS analysis was performed after 15 h of coculture. Cytotoxicity was assessed by Fixable Viability Dyes (FVD) eFluor™ 780 (eBioscience). The percentage of FVD 780^+^ cells, gated on CFSE^+^ cells, was measured as the indicator of NK cell-mediated cytoxicity.

### In vitro stimulation assays

PBMCs (1 × 10^6^ cells/ml) and sorted NK cells were treated with hydrogen peroxide(H_2_O_2_) at concentrations of 20 µM, 40 µM and 80 µM, respectively, for 24 h culture in RPMI 1640 medium (10% FBS) at 37℃ in a 5% CO_2_ atmosphere. H_2_O_2_ was diluted in PBS and used within minutes of preparation.

### T-distributed stochastic neighbour embedding (t-SNE) analysis

Samples (from four SLE patients with active disease activities and four HCs) were used for t-SNE analysis using R scripts based on the ‘flowCore’ package and ‘Rtsne’ package [16]. In R, all data were transformed using the logicleTransform function to roughly match scaling historically used in FlowJo.

### Gene Set Enrichment Analysis (GSEA)

The gene expression datasets were attained from the Gene Expression Omnibus (GEO) database (http://www.ncbi.nlm.nih.gov/geo/)(GSE63829 and GSE23695). And datasets were analyzed using R scripts based on the ‘clusterProfiler’ package [17]. Normalized enrichment score (NES) was calculated for every gene set (using p value < 0.05 and FDR < 0.25 as the threshold). The gene sets used in the GSEA belong to hallmark gene sets from Molecular Signature v7.2 Database (MSigDB).

### Statistical analysis

Continuous variables were expressed as mean ± SD or median(interquartile range, IQR). Categorical variables were expressed as number(percent). The chi-square test or Fisher test was used to compare qualitative values according to distribution. The Student’s unpaired or paired t test was performed to compare two groups for parametric data, and the Mann–Whitney U test or Wilcoxon rank sum test was performed for nonparametric data. The Pearson test or Spearman was used to determine the correlation between variables. The statistical analyses were performed using R v.3.6.1 software. And the figures were drawn using GraphPad Software. P values less than 0.05 were considered significant.

## Results

### Peripheral NK cell deficiency was associated with disease activity in treatment-naive SLE patients

**Fig. 1:**
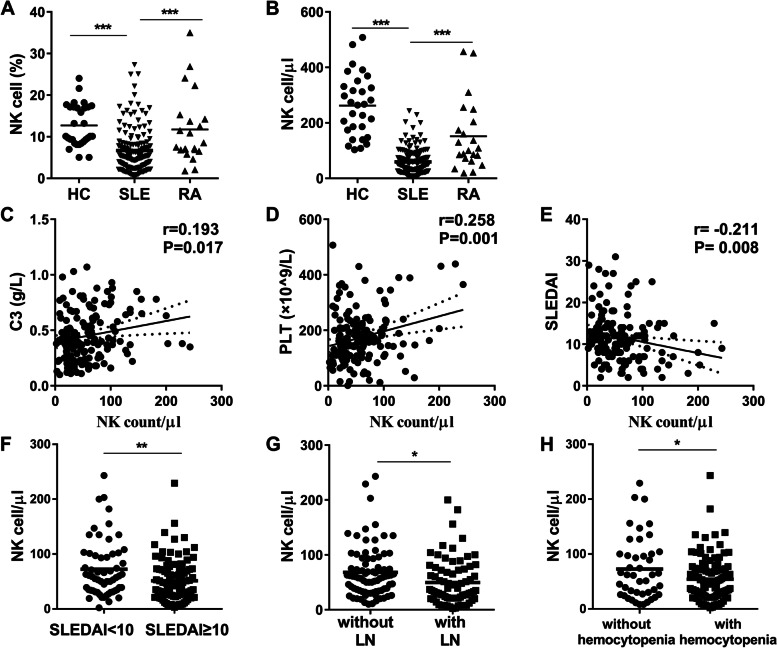
Peripheral NK cell deficiency was associated with disease activity in treatment-naive SLE patients. **A** The percentage of NK cells in untreated SLE patients (*n* = 156), untreated RA patients (*n* = 22) and HCs (*n* = 30). **B** The absolute number of peripheral NK cells in untreated SLE patients (*n* = 156), untreated RA patients (*n* = 22) and HCs (*n* = 30). **C** Correlation of the NK count with the level of C3 in SLE patients. **D** Correlation of the NK count with the level of PLT in SLE patients. **E** Correlation of the NK count with SLEDAI score in SLE patients. **F** Comparison of the absolute number of peripheral NK cells in SLE patients with mild activity (SLEDAI < 10) (*n* = 97) and moderate-high activity (SLEDAI ≥ 10) (*n* = 59). **G** Comparison of the absolute number of peripheral NK cells in SLE patients with (*n* = 74) and without LN (*n* = 82). **H** Comparison of the absolute number of peripheral NK cells in SLE patients with (*n* = 112) and without hemocytopenia (*n* = 44). (* *P* < 0.05, **P < 0.01, ***P < 0.001). C3: serum concentrations of complement C3; PLT: blood platelet; SLEDAI: Systemic Lupus Erythematosus Disease Activity Index; LN: lupus nephritis

The present study included a total of 156 treatment-naive SLE patients (median age 33, IQR:26–47 years, 90.4% female), 22 treatment-naive RA patients (median age 37, IQR: 32–47 years, 90.9% female) and 30 HCs (median age 32, IQR:27–38 years, 90.0% female). The demographic and clinical manifestations of these participants were shown in Supplementary Table 2. The percentages and counts of NK cells were remarkably down-regulated in untreated SLE patients compared with that of untreated RA patients or HCs [NK cell percentage: 6.68 ± 5.17%,11.75 ± 8.46% and 12.72 ± 5.04% respectively; NK cell count: 50 (26–77) /µl, 104 (70–215) /µl and 266 (167–336) /µl respectively, Fig. 1A, B, all *P* < 0.001], suggesting that NK cell deficiency was associated with the disease itself regardless of treatment. While there was no significant difference in CD56 MFI of NK cells between SLE patients and HCs (Supplementary Fig. 2A).

Correlation analysis showed that in treatment-naive SLE patients, the NK count showed weak positive correlations with the levels of complement C3 (*r* = 0.193, *P* = 0.017) (Fig. 1C) and PLT (*r* = 0.258, *P* = 0.001) (Fig. 1D) and a negative correlation with SLEDAI score (*r* =  − 0.211, *P* = 0.008) (Fig. 1E). No significant correlations were found between peripheral NK count and the levels of anti-dsDNA antibody and complement C4 (Supplementary Fig. 2B, C). In addition, no significant correlation was found between peripheral NK count with the level of DAS28 in RA patients (*r* = 0.03, *P* = 0.89) (Supplementary Fig. 2D). SLE patients were grouped based on their SLEDAI score: mild activity (SLEDAI < 10) and moderate-high activity (SLEDAI ≥ 10). SLE patients in moderate-high activity group showed lower NK count [45 (21–72) /µl, *n* = 97 versus 59 (33–100) /µl, *n* = 59, *P* < 0.01, Fig. 1F]. Compared to SLE patients without LN [59 (33–93) /µl, *n* = 82], NK count was lower in SLE patients with LN [35 (21–72) /µl, *n* = 74] (*P* < 0.05) (Fig. 1G). NK count was lower in SLE patients with hemocytopenia [48 (26–72) /µl, *n* = 112] compared to SLE patients without hemocytopenia [61 (26–100) /µl, *n* = 44] (*P* < 0.05) (Fig. 1H). There were no statistic differences between two groups when grouped by rash, arthritis and central nervous system (CNS) involvement (Supplementary Fig. 2E-G).

### Peripheral CD56^dim^CD57^+^ NK cells exhibited a selective reduction in SLE patients

**Fig. 2:**
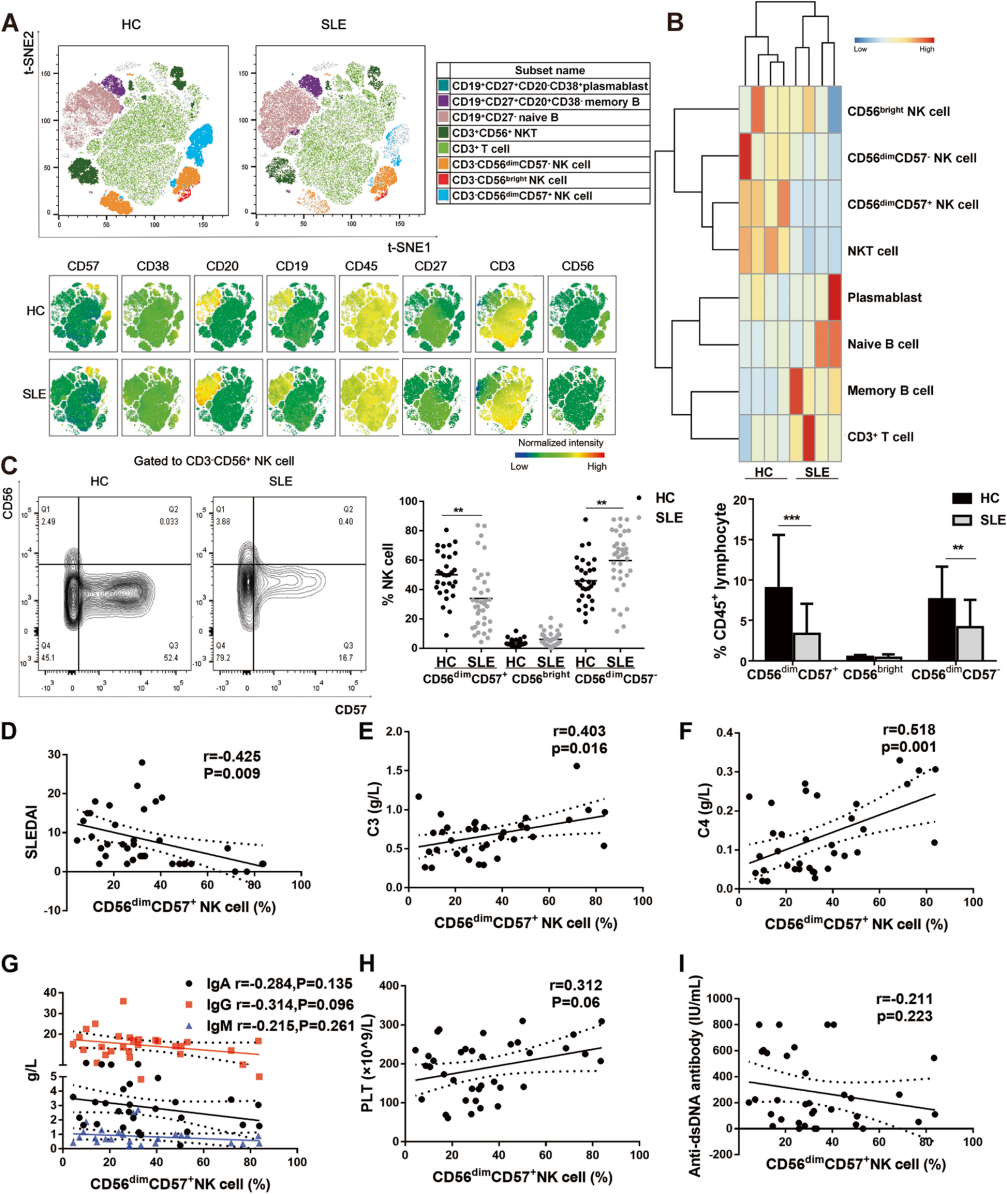
Peripheral CD56^dim^CD57^+^ NK cells exhibited a selective reduction in SLE patients. **A** t-SNE map of CD45^+^ lymphocyte cells from HCs and active SLE patients. There were 8 subsets (CD3^−^CD19^+^CD27^+^CD20^−^CD38^+^ plasmablast, CD3^−^CD19^+^CD27^+^CD20^+^CD38^−^ memory B, CD3^−^CD19^+^CD27^−^ naive B, CD3^+^CD56^+^ NKT, CD3^+^T cell, CD3^−^CD56^dim^CD57^−^ NK cell, CD3^−^CD56^bright^ NK cell, and CD3^−^CD56^dim^CD57^+^ NK cell). t-SNE was performed simultaneously on four SLE patients with active disease activities and four HCs. **B** Heatmap representing scaled expression values of the 8 subsets between HC (*n* = 4) and SLE patients (*n* = 4). **C** Representative flow cytometric plot for NK cell subsets (including CD3^−^CD56^dim^CD57^+^ NK cells, CD3^−^CD56^bright^ NK cells, and CD3^−^CD56^dim^CD57^−^ NK cells) from a HC and a SLE patient (Left panel). The percentage of three NK cell subsets in total NK cells from patients (*n* = 37) and HCs (*n* = 30) (Middle panel). The proportion of NK cell subsets in CD45^+^ lymphocytes in SLE patients (*n* = 37) and HCs (*n* = 30) (Right panel). **D** The correlation of CD56^dim^CD57^+^ NK cell proportion with SLEDAI score. **E** The correlation of CD56^dim^CD57^+^ NK cell proportion with the level of C3. **F** The correlation of CD56^dim^CD57^+^ NK cell proportion with the level of C4. **G** The correlations of CD56^dim^CD57^+^ NK cell proportion with the levels of IgG, IgM and IgA. **H** The correlation of CD56^dim^CD57^+^ NK cell proportion with the level of PLT. **I** The correlation of CD56^dim^CD57^+^ NK cell proportion with the level of anti-dsDNA antibody. (* *P* < 0.05, **P < 0.01, ***P < 0.001). tSNE: t-distributed stochastic neighbour embedding; SLEDAI: Systemic Lupus Erythematosus Disease Activity Index;C3: serum concentrations of complement C3; C4: serum concentrations of complement C4; IgG:immunoglobulin G; IgM:immunoglobulin M; IgA:immunoglobulin A;PLT: blood platelet; anti-dsDNA antibody: anti-double-stranded-DNA-specific antibody

We described a t-SNE map of CD45^+^ lymphocyte cells on four SLE patients with active disease activities and four HCs. The t-SNE map (Fig. 2A) and heatmap (Fig. 2B) visually showed an abnormal distribution of subpopulations [18] between SLE patients and HCs. Compared with HCs, SLE patients exhibited fewer NK cell subsets (Fig. 2A-B), especially for CD56^dim^CD57^+^ NK subgroup. To further confirm the above results and assess the relationship between NK subsets and disease activities, three NK cell subpopulations (CD56^bright^ NK cells, CD56^dim^CD57^−^ NK cells, and CD56^dim^CD57^+^ NK cells; gating strategy shown in Supplementary Fig. 1) were studied in 37 untreated SLE patients and 30 HCs (Fig. 2C). Our study indicated that SLE patients exhibited a selective reduction in CD56^dim^CD57^+^ NK cells (33.9 ± 21.5% versus 50.1 ± 15.8%, *P* < 0.01), but not CD56^bright^ NK cells (6.09 ± 4.88% versus 3.57 ± 2.44%) or CD56^dim^CD57^−^ NK cells (59.8 ± 20.2% versus 46.1 ± 15.5%) compared with HCs (Fig. 2C, middle panel). Instead, CD56^dim^CD57^−^ NK cell proportion was significantly increased in SLE patients (*P* < 0.01). The proportions of three NK cell subsets in CD45^+^ lymphocytes from SLE patients were decreased (CD56^dim^CD57^+^ NK cell percentage: 9.01 ± 6.56% and 3.32 ± 3.73% for HCs and SLE respectively; CD56^bright^ NK cell percentage: 0.49 ± 0.21% and 0.39 ± 0.39% for HCs and SLE respectively; CD56^dim^CD57^−^ NK cell percentage: 7.60 ± 4.04% and 4.15 ± 3.38% for HCs and SLE respectively), and the two groups of CD56^dim^ NK cells reached the statistically significant differences (*P* < 0.01) (Fig. 2C, right panel).

Furthermore, correlation analysis showed that CD56^dim^CD57^+^ NK cell proportion was negatively correlated with SLEDAI score (*r* =  − 0.425, *P* = 0.009) (Fig. 2D), positively correlated with levels of complement C3 (*r* = 0.403, *P* = 0.016) and C4 (*r* = 0.518, *P* = 0.001) (Fig. 2E, F). In addition, the levels of PLT and anti-dsDNA antibody were weakly associated with CD56^dim^CD57^+^ NK cell proportion (Fig. 2G-I). No significant correlation was found between CD56^bright^ NK cell proportion and SLEDAI score (*r* = 0.107, *P* = 0.52) (Supplementary Fig. 3).

Together, these findings demonstrated that SLE patients had not only a global reduction in their peripheral NK cells but also an alteration in subpopulations of NK cells-a loss of mature CD56^dim^CD57^+^ NK cells which were negatively correlated with disease activities.

### Peripheral CD56^dim^CD57^+^ NK cells underwent increased endogenous apoptosis in SLE patients

**Fig. 3:**
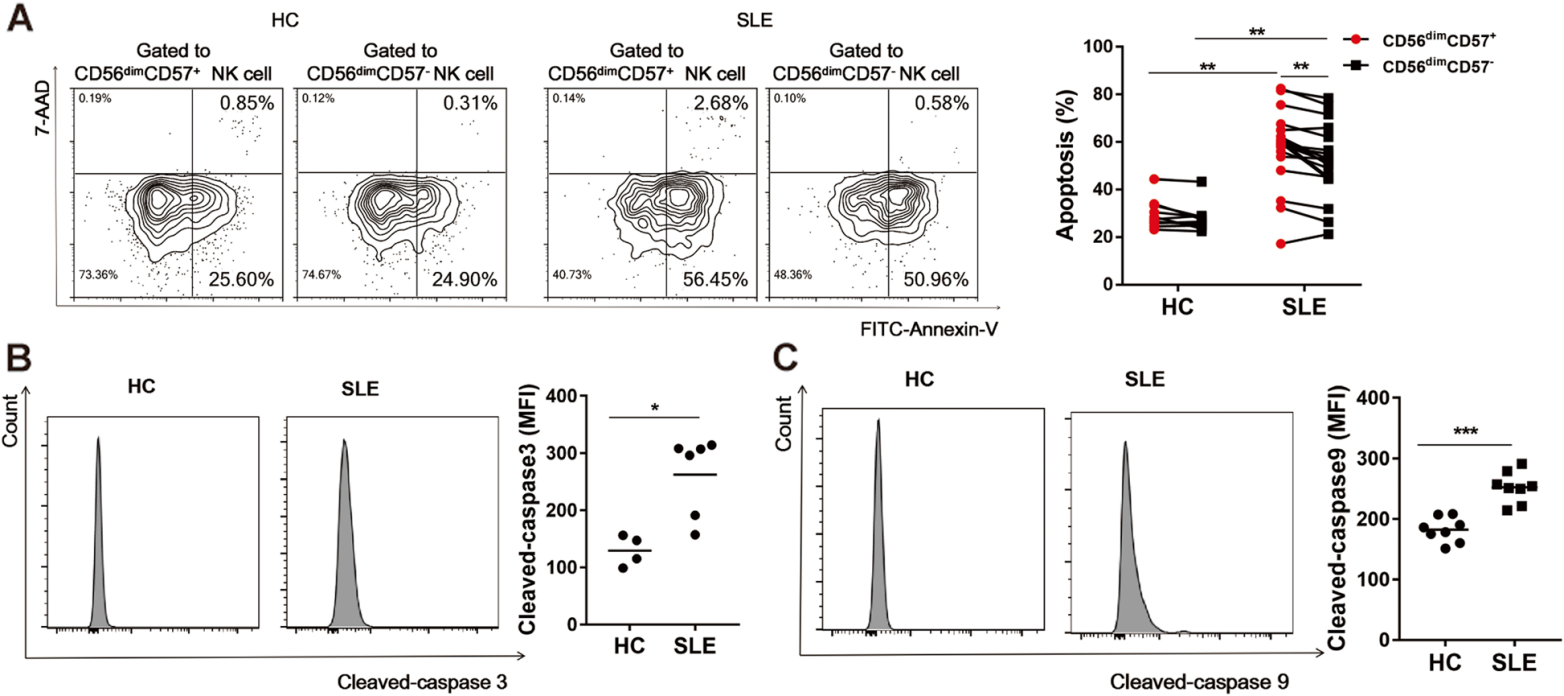
Peripheral CD56^dim^CD57^+^ NK cells underwent increased endogenous apoptosis in SLE patients. **A** Representative flow cytometric plot for apoptosis of NK cells (CD56^dim^CD57^+^ NK cell subset and CD56^dim^CD57^−^ NK cell subset) in one HC and one SLE patient (Left panel). Comparison of total apoptotic rate of NK cell subsets between HCs (*n* = 10) and SLE patients (*n* = 19) (Right panel). **B** Comparison of Cleaved-caspase 3 level of CD56^dim^CD57^+^ NK cells in SLE patients (*n* = 6) and HCs (*n* = 4). **C** Comparison of Cleaved-caspase 9 level of CD56^dim^CD57^+^ NK cells in SLE patients (*n* = 8) and HCs (*n* = 8). (* *P* < 0.05, **P < 0.01, ***P < 0.001)

To delineate the underlying mechanisms for the apparent reduction of peripheral NK cells in SLE patients, Annexin-V/7-AAD double staining flow cytometry was applied to detect the proportion of apoptotic NK cells. Total apoptotic rate of peripheral CD56^dim^CD57^+^ NK cells (57.7 ± 16.0%, *n* = 19 versus 29.8 ± 6.2%, *n* = 10, *P* < 0.01) and CD56^dim^CD57^−^ NK cells (52.0 ± 15.2%, *n* = 19 versus 27.9 ± 5.8%, *n* = 10, *P* < 0.01) in SLE patients were both increased compared with that in HCs (Fig. 3A). Total apoptotic rate of peripheral CD56^dim^CD57^+^ NK cells in SLE patients was increased compared with CD56^dim^CD57^−^ NK cells in SLE patients (57.7 ± 16.0%, *n* = 19 versus 52.0 ± 15.2%, *n* = 19, *P* < 0.01) (Fig. 3A).

Cleaved-caspase 3 is an important regulator of apoptosis and Cleaved-caspase 9 mediates endogenous apoptosis. Compared with CD56^dim^CD57^+^ NK cells in HCs, CD56^dim^CD57^+^ NK cells in SLE patients showed increased expressions of Cleaved-caspase 3 (MFI: 129 ± 26.8, *n* = 4 versus 238 ± 75.3, *n* = 6, *P* < 0.05, Fig. 3B) and Cleaved-caspase 9 (MFI: 181 ± 20.3, *n* = 8 versus 262 ± 69.3, *n* = 8, *P* < 0.001, Fig. 3C).

Collectively, the selective reduction in CD56^dim^CD57^+^ NK cells was due to, at least in part, increased endogenous apoptosis level in lupus microenvironment.

### Peripheral CD56^dim^CD57^+^ NK cells showed higher oxidative stress in SLE patients

**Fig. 4:**
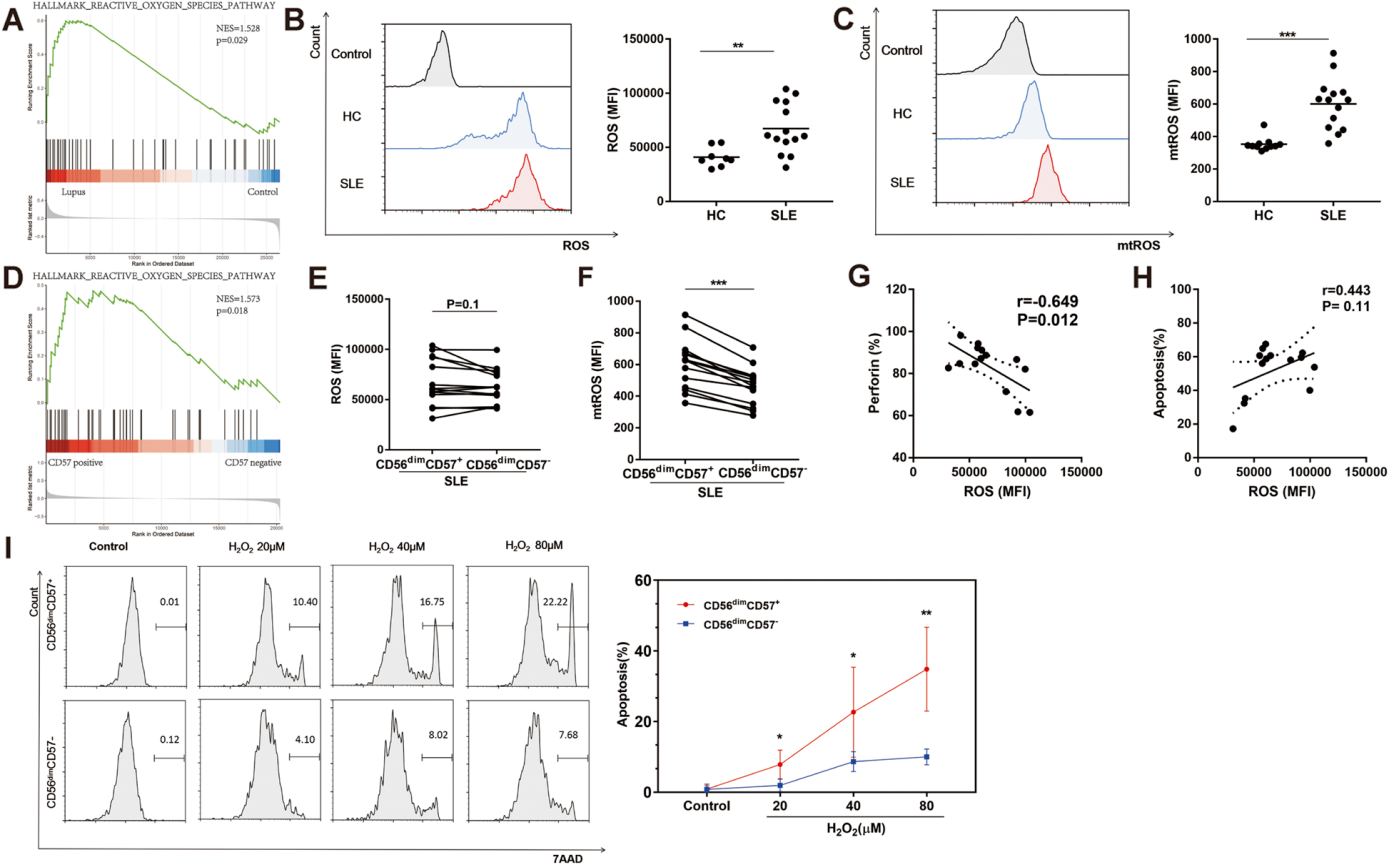
Peripheral CD56^dim^CD57^+^ NK cells showed higher oxidative stress in SLE patients. **A** GSEA plots showing an increase in ROS signature (enrichment plot: HALLMARK_REACTIVE_OXYGEN_SPECIES_PATHWAY) in lupus NK cells compared with normal NK cells (GSE63829). **B** Production of ROS in CD56^dim^CD57^+^NK cells from SLE patients (*n* = 14) and HCs (*n* = 8). **C** Production of mtROS in CD56^dim^CD57^+^NK cells from SLE patients (*n* = 14) and HCs (*n* = 11). **D** GSEA plots showing an increase in ROS signature (enrichment plot: HALLMARK_REACTIVE_OXYGEN_SPECIES_PATHWAY) in CD57 + NK cells compared with CD57-NK cells (GSE23695). **E** Comparison of ROS level of CD56^dim^CD57^+^ NK cell subset and CD56^dim^CD57^−^ NK cell subset in SLE patients (*n* = 14). **F** Comparison of mtROS level of CD56^dim^CD57^+^ NK cell subset and CD56^dim^CD57^−^ NK cell subset in SLE patients (*n* = 14). **G** The correlation of ROS level and perforin expression in CD56^dim^CD57^+^ NK cell subset in SLE patients(*n* = 14). **H** The correlation of ROS level and total apoptosis in CD56^dim^CD57^+^ NK cell subset in SLE patients (*n* = 14). **I** Comparison of apoptosis level of CD56^dim^CD57^+^ NK cell subset and CD56^dim^CD57^−^ NK cell subset from HC upon exposure to H_2_O_2_ for 24 h culture at concentrations of 20 µM, 40 µM and 80 µM, respectively (*n* = 5). GSEA: Gene set enrichment analysis; ROS: reactive oxygen species; mtROS: mitochondrial reactive oxygen species. (* *P* < 0.05, **P < 0.01, ***P < 0.001)

To identify cellular programs that may underlie low levels of NK cell activity in lupus, we analyzed a published dataset [19] (GSE63829) by GSEA for Hallmark gene sets, which are coherently expressed signatures derived by aggregating many MSigDB gene sets to represent well-defined biological states or processes. Since there was no appropriate data of human NK cells, we adopted GSE63829 from lupus prone mouse. Among 50 hallmark gene sets from MSigDB, ROS pathway was significantly enriched (NES = 1.528, *p* = 0.029, Fig. 4A) in NK cells from lupus, compared with controls. All enriched pathways were summarize in Supplementary Table 3. As mitochondria are key cellular sources of ROS, total and mitochondrial ROS levels were assessed in NK cells. Both ROS (67,357 ± 23,137, *n* = 14 versus 40,983 ± 9046, *n* = 8, *P* < 0.01, Fig. 4B) and mtROS ( 601 ± 157, *n* = 14 versus 353 ± 42.0, *n* = 11, *P* < 0.001, Fig. 4C) levels of CD56^dim^CD57^+^ NK cells were significantly increased in SLE patients.

Interestingly, ROS pathway was highly activated (NES = 1.573, *p* = 0.018, Fig. 4D and Supplementary Table 4) in CD56^dim^CD57^+^ NK cells compared with the CD56^dim^CD57^−^ NK cell subset when we analyzed another published dataset [6] (GSE23695). The two subsets in GSE23695 are CD3^−^CD56^dim^CD16^+^CD57^+^ and CD3^−^CD56^dim^CD16^+^CD57^−^ NK cells. In peripheral blood of healthy people, CD56^dim^ NK cells express high levels of CD16. So we use that data for our study comparing CD56^dim^CD57^+^ and CD56^dim^CD57^−^ NK cells. Interestingly, ROS pathway was enriched in both GSE63829 and GSE23695. In addition, ROS level (67,357 ± 23,137, *n* = 14 versus 63,539 ± 16,767, *n* = 14, *P* = 0.1, Fig. 4E) was marginally higher and mtROS level (601 ± 157, *n* = 14 versus 464 ± 119, *n* = 14, *P* < 0.001, Fig. 4F) was much higher in CD56^dim^CD57^+^ NK cells in SLE patients compared with CD56^dim^CD57^−^ NK cells. Of note, we observed a strong negative association between ROS level and perforin expression (*r* =  − 0.649, *P* = 0.012, Fig. 4G), and a weak positive association between ROS level and total apoptosis (*r* = 0.443, *P* = 0.11, Fig. 4H) in CD56^dim^CD57^+^ NK cells from SLE patients. These indicators reflected a higher oxidative stress of CD56^dim^CD57^+^ NK cells in SLE patients, which may influence NK cell activities.

Therefore, we next performed a detailed analysis aiming to reveal the effect of ROS on the two NK subsets. Total PBMCs were treated with different concentrations of H_2_O_2_ to mimic physiological levels of ROS exposure [20]. With increased concentrations of H_2_O_2_, apoptotic cells were increased in both two NK cell subsets (Fig. 4I). At concentrations of 20 µM and above, apoptotic rates of CD56^dim^CD57^+^ NK cells were higher than CD56^dim^CD57^−^ NK cells when they were exposed to the same H_2_O_2_ level, suggesting that CD56^dim^CD57^+^ NK cells were more prone to undergo apoptosis when exposed to ROS (Fig. 4I). To confirm this finding more clearly, we sorted NK cells from PBMCs. Consistent with the data from PBMCs, our results verified that apoptotic rates of CD57^+^ NK cells were higher than CD57^−^ NK cells at the same H_2_O_2_ level (Supplementary Fig. 4A). What is more, ROS levels of CD57^+^ NK cells were higher than CD57^−^ NK cells when they were exposed to the same H_2_O_2_ level (Supplementary Fig. 4B).

These studies suggested that CD56^dim^CD57^+^ NK cells were preferentially targeted for oxidative stress in SLE patients, leading to decreased activity of this NK subset.

### Peripheral CD56^dim^CD57^+^ NK cells exhibited attenuated cytotoxicity and altered phenotypes in SLE patients

The distinctive characteristics of CD56^dim^ NK cells and CD56^bright^ NK cells have been well-characterized, and the former are predominant in the peripheral blood, with stronger cytotoxic function. To assess the functions of two CD56^dim^ NK cell subsets (CD56^dim^CD57^+^ NK cells and CD56^dim^CD57^−^ NK cells), we examined the markers associated with degranulation, activation and cytokine production.

**Fig. 5:**
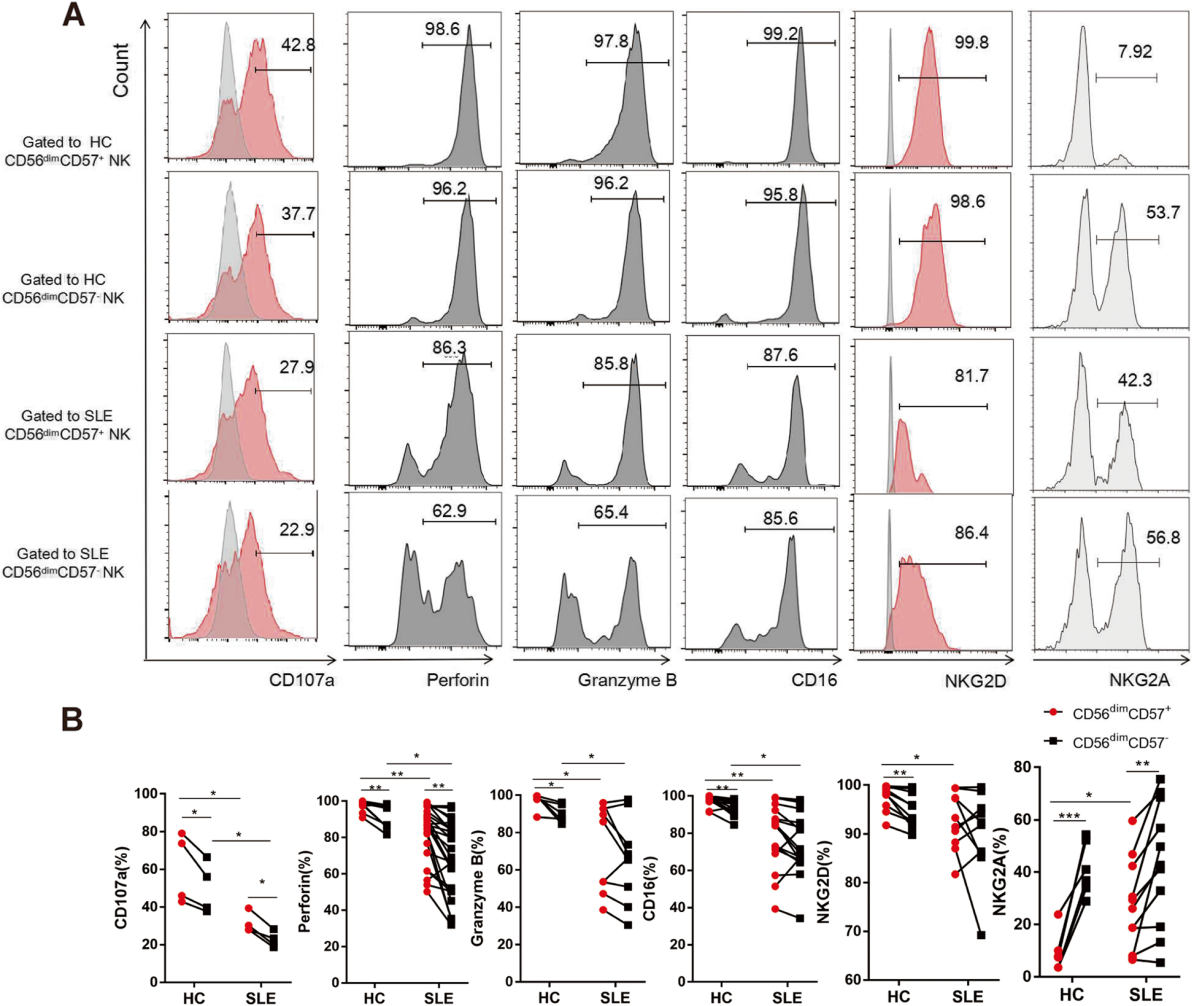
Peripheral CD56^dim^CD57^+^ NK cells exhibited attenuated cytotoxicity and altered phenotypes in SLE patients. **A** Representative flow cytometric plot for expression of CD107a, perforin, Granzyme B, CD16, NKG2D and NKG2A on NK cell subsets (CD56^dim^CD57^+^ NK cell subset and CD56^dim^CD57^−^ NK cell subset) in HC and SLE patient. **B** Comparison of CD107a, perforin, Granzyme B, CD16, NKG2D and NKG2A expression on NK cell subsets. (* *P* < 0.05, **P < 0.01, ***P < 0.001)

In our research, flow cytometry analysis confirmed the discrepancy of CD56^dim^CD57^+^ NK cells and CD56^dim^CD57^−^ NK cells. The expression of CD107a on the cell membrane correlates with NK cell killing activity. Compared with CD56^dim^CD57^−^ NK cells, CD56^dim^CD57^+^ NK cells exhibited higher expression of CD107a, perforin, granzyme B, CD16 and active receptor NKG2D in HCs (CD107a: 60.4 ± 18.6% versus 49.9 ± 13.7%, *n* = 4; perforin: 97.5 ± 3.01% versus 92.2 ± 6.50%, *n* = 10; granzyme B: 97.3 ± 4.47% versus 90.0 ± 4.69%, *n* = 6; CD16: 97.7 ± 3.01% versus 93.7 ± 4.30%, *n* = 12; NKG2D: 97.5 ± 2.65% versus 94.8 ± 3.49%, *n* = 10, Fig. 5A, B, all *P* < 0.05). With respect to inhibitory receptors specific for MHC class I, NKG2A expression was decreased on CD56^dim^CD57^+^ NK cells relative to that on CD56^dim^CD57^−^ NK cells in HCs (8.54 ± 7.20% versus 42.9 ± 10.4%, *n* = 7, *P* < 0.001, Fig. 5A, B). The expression of IFN-γ (25.7 ± 19.4% versus 36.4 ± 16.1%, *n* = 5) and IL-10 (5.27 ± 3.17% versus 5.14 ± 2.51%, *n* = 5) did not differ between CD56^dim^CD57^+^ NK cells and CD56^dim^CD57^−^ NK cells in HCs (Supplementary Fig. 5). The discrepancies between CD56^dim^CD57^+^ NK cells and CD56^dim^CD57^−^ NK cells in SLE patients were similar (CD107a: 31.3 ± 5.46% versus 22.5 ± 4.30%, *n* = 4; perforin: 82.2 ± 15.1% versus 69.1 ± 20.1%, *n* = 24; granzyme B: 72.5 ± 23.6% versus 65.3 ± 22.6%, *n* = 9; CD16: 79.3 ± 17.5% versus 75.2 ± 16.8%, *n* = 17; NKG2D: 92.5 ± 5.90% versus 89.8 ± 8.87%, *n* = 10; NKG2A: 26.7 ± 17.3% versus 43.2 ± 23.8%, *n* = 11; IFN-γ: 16.3 ± 10.6% versus 24.1 ± 8.19%, *n* = 7; IL-10: 8.51 ± 3.64% versus 10.5 ± 3.82%, *n* = 6, Fig. 5A, B), but to a lesser extent than that in HCs. Our research demonstrated that CD56^dim^CD57^+^ NK cells exhibited enhanced cytotoxic function.

We also observed a significant impairment in CD107a expression by CD56^dim^CD57^+^ NK cells from SLE patients relative to that from HCs (31.3 ± 5.46%, *n* = 4 versus 60.4 ± 18.6%, *n* = 4, *P* < 0.05, Fig. 5A, B). Compared with CD56^dim^CD57^+^ NK cells in HCs, CD56^dim^CD57^+^ NK cells in SLE patients showed decreased expressions of perforin (97.5 ± 3.01%, *n* = 10 versus 82.2 ± 15.1%, *n* = 24, *P* < 0.01), granzyme B (97.3 ± 4.47%, *n* = 6 versus 72.5 ± 23.6%, *n* = 9, *P* < 0.05), CD16 (97.7 ± 3.01%, *n* = 12 versus 79.3 ± 17.5%, *n* = 17, *P* < 0.01) and NKG2D (97.5 ± 2.65%, *n* = 10 versus 92.5 ± 5.90%, *n* = 10, *P* < 0.05), along with increased NKG2A expression (8.54 ± 7.20%, *n* = 7 versus 26.7 ± 17.3%, *n* = 11, *P* < 0.05) (Fig. 5A, B). There were no significant differences between the two groups in terms of IFN-γ (25.7 ± 19.4%, *n* = 5 versus 16.3 ± 10.6%, *n* = 7) and IL-10 (5.27 ± 3.17%, *n* = 5 versus 8.51 ± 3.64%, *n* = 6) expression (Supplementary Fig. 5). In CD56^dim^CD57^−^ NK cells, there were differences in percentage of CD107a (49.9 ± 13.7%, *n* = 4 versus 22.5 ± 4.30%, *n* = 4, *P* < 0.05), perforin (92.2 ± 6.50%, *n* = 10 versus 69.1 ± 20.1%, *n* = 24, *P* < 0.05), granzyme B (90.0 ± 4.69%, *n* = 6 versus 65.3 ± 22.6%, *n* = 9, *P* < 0.05) and CD16 (93.7 ± 4.30%, *n* = 12 versus 75.2 ± 16.8%, *n* = 17, *P* < 0.05) between HCs and SLE patients, but no differences in expressions of NKG2D and NKG2A (Fig. 5A, B).

Together, CD56^dim^CD57^+^ NK cells in SLE patients exhibited not only lower proportion but also impaired in vitro function, especially the cytotoxic function.

### Peripheral CD56^dim^CD57^+^ NK cells from SLE patients exhibited impaired cytotoxicity in response to activated CD4^+^ T cells

**Fig. 6:**
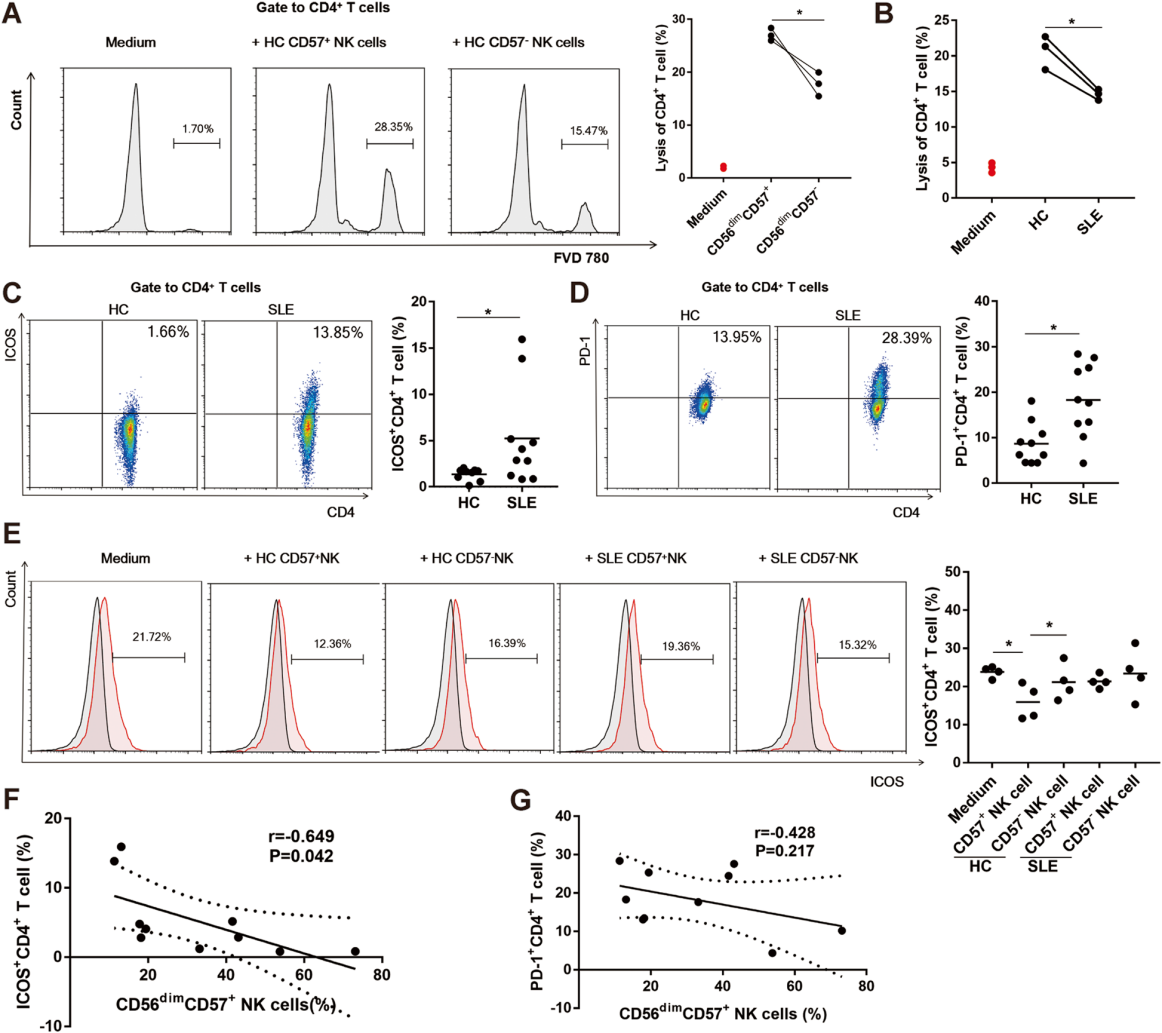
Peripheral CD56^dim^CD57^+^ NK cells from SLE patients exhibited impaired cytotoxicity in response to activated CD4^+^ T cells. **A** Cytoxicity assay of CD4^+^ T cells in response to NK cell subsets (*n* = 3). **B** Cytoxicity assay of CD4^+^ T cells in response to CD56^dim^CD57^+^ NK cells from SLE patients and HCs (*n* = 3). **C** Comparison of ICOS expression on CD4^+^ T cells from SLE patients (*n* = 10) and HCs (*n* = 9). **D** Comparison of PD-1 expression on CD4^+^ T cells from SLE patients (*n* = 10) and HCs (*n* = 10). **E** Comparison of ICOS expression on CD4^+^ T cells after co-culture with NK subsets (*n* = 4). **F** The correlation of ICOS expression on CD4^+^ T cells and CD56^dim^CD57^+^ NK cell percentage in SLE patients (*n* = 10). **G** The correlation of PD-1 expression on CD4^+^ T cells and CD56^dim^CD57^+^ NK cell percentage in SLE patients (*n* = 10). ICOS: Inducible T cell costimulator; PD-1: programmed death 1. (* *P* < 0.05)

Activated CD4^+^ T cells are involved in multiple aspects of SLE pathogenesis [1]. NK cells can negatively regulate CD4^+^ T cell responses to protect from immunopathology, and most reports have found that this process is dependent on direct cytotoxicity [3, 4]. We observed specific lysis of autologous activated CD4^+^ T cells by NK cells, but no killing of resting CD4^+^ T cells (Fig. 6A, Supplementary Fig. 6), which is consistent with the findings of other investigators [4, 21]. In addition, sorted CD56^dim^CD57^+^ NK cells manifested significantly enlarged cytotoxicity relative to CD56^dim^CD57^−^ NK cells in response to autologous activated CD4^+^ T cells from HCs (27.1 ± 1.19%, *n* = 3 versus 17.7 ± 2.24%, *n* = 3, *P* < 0.05, Fig. 6A). As there are differences in CD4^+^ T cells between HCs and SLE patients, we chose the same allogeneic CD4^+^ T cell as the target cells. CD56^dim^CD57^+^ NK cells from SLE patients showed significantly attenuated cytotoxicity compared with that from HCs when co-cultured with the same allogeneic activated CD4^+^ T cells (14.5 ± 0.75%, *n* = 3 versus 20.7 ± 2.38%, *n* = 3, *P* < 0.05, Fig. 6B).

On CD4^+^ T cells, inducible T cell costimulator (ICOS) and programmed death 1 (PD-1) are activation markers which play critical roles in B cell activation and differentiation [1, 22]. Our study also confirmed that both the percentages of ICOS (5.24 ± 5.04%, *n* = 10 versus 1.36 ± 0.64%, *n* = 9, *P* < 0.05) and PD-1 (18.3 ± 8.13%, *n* = 10 versus 8.71 ± 4.65%, *n* = 10, *P* < 0.05) on CD4^+^ T cells were higher in SLE patients than in HCs (Fig. 6C, D). In addition, there was no statistical difference in PD-1 expression in NK cell subsets between the two groups (Supplementary Fig. 7). The expression of ICOS on CD4^+^ T cells decreased after co-culture with CD57^+^ NK cells from HCs (23.8 ± 1.49%, *n* = 4 versus 15.9 ± 4.64%, *n* = 4), and such an inhibitory effect was not significant for NK cell subsets from SLE patients (Fig. 6E). We further tested whether NK cell subset distribution was correlated with activation makers of CD4^+^ T cells in SLE. Interestingly, lower percentage of CD56^dim^CD57^+^ NK cells strongly correlated with higher expression of active maker ICOS (*r* =  − 0.649, *P* = 0.042, Fig. 6F). We also observed a trend of, though not statistically significant, negative correlation between CD56^dim^CD57^+^ NK cell percentage and PD-1 expression (*r* =  − 0.428, *P* = 0.217, Fig. 6G). The above results suggested a regulatory effect of NK cell subset distribution on activation of CD4^+^ T cells.

These data indicated that NK cells, especially CD56^dim^CD57^+^ NK cells, targeted activated CD4^+^ T cells. The selective reduction in CD56^dim^CD57^+^ NK cells and impaired function in SLE patients might impede their ability to eradicate activated pathogenic CD4^+^ T cells which are key cells that cause lupus disease.

## Discussion

In this study, we demonstrated that the number and percentage of NK cells decreased substantially in the peripheral blood of treatment-naive SLE patients compared with those of HCs and treatment-naive RA patients. Beyond reduced numbers, NK cells in SLE patients had a selective loss of mature CD56^dim^CD57^+^ NK cell subset, which was conversely correlated with disease activity. CD56^dim^CD57^+^ NK cells exhibited increased cytotoxic effect on activated CD4^+^ T cells. Additionally, attenuated cytotoxicity function, up-regulated endogenous apoptosis and increased oxidative stress were observed in peripheral CD56^dim^CD57^+^ NK cells from SLE patients.

Previous studies have shown significantly down-regulated counts and abnormal phenotypes of peripheral NK cells in SLE patients [10–12]. To exclude the influence of glucocorticoids or immunosuppressive drugs, we enrolled treatment-naive patients. We identified an extremely reduced NK count (median 50 /µL) in SLE patients, which was less than a quarter of HCs (median 266 /µL). Our study confirmed that compared with both HCs and untreated RA patients, numbers and percentages of NK cells were remarkably down-regulated in SLE patients, suggesting that NK cell deficiency was mainly due to immune disorder underlying lupus itself regardless of treatment. Our study also indicated that SLE patients exhibited a selective reduction in CD56^dim^CD57^+^ NK cells which was negatively correlated with SLEDAI, positively correlated with both levels of complement C3 and C4, and weakly associated with anti-dsDNA antibody level. Our research suggests that CD56^dim^CD57^+^ NK cell deficiency might be an important intrinsic mechanism for the pathogenesis of SLE.

Beyond being important innate immune cells, NK cells may regulate adaptive immune responses in many aspects [3, 4, 23]. In vivo studies showed that, through a perforin-dependent pathway, NK cells can not only directly eliminate activated CD4^+^ T cells but also exhibit potent immunosuppressive capacity that repress antigen-specific B cell and immunoglobulin somatic mutations [3, 23]. Activated CD4^+^ T cells may express cell-surface NKG2D ligands and become susceptible to autologous NK-cell lysis [24, 25]. NK cell depletion can lead to the enhanced development of experimental autoimmune encephalomyelitis (EAE) due to increased numbers of auto-reactive CD4^+^ T cells [26]. Hemophagocytic lymphohistiocytosis (HLH), a disease linked to genetic defects in perforin mediated cytotoxicity, has been associated with impaired NK cells and increased proliferation of T cells [27]. The immunologic hallmark of SLE patients is the breakdown of tolerance, manifested as CD4^+^ T cell activation, B cell amplification and autoantibody production [1, 22]. ICOS is a crucial activation marker of CD4^+^ T cells and plays critical roles in T-B interaction for B cell activation and differentiation [22, 28]. Therefore, ICOS positive CD4^+^ T cells might be pathogenic in SLE. Our study confirmed that the expression of ICOS on CD4^+^ T cells was particularly low in healthy controls while significantly increased in SLE patients. In the co-culture experiments, the expression of ICOS on CD4^+^ T cells decreased after co-culture with NK cells, and such an inhibitory effect was more significant for CD57^+^ NK cells from healthy controls than those from SLE patients. Furthermore, we observed lysis of activated CD4^+^ T cells, but not resting CD4^+^ T cells, when cocultured with NK cells. In addition, CD56^dim^CD57^+^ NK cells exhibited stronger cytotoxicity in response to activated CD4^+^ T cells relative to CD56^dim^CD57^−^ NK cells. Our research demonstrated that two CD56^dim^ NK cell subsets exhibited lower expression of CD107a, perforin and granzyme B in SLE patients. Whlie the single-cell RNA-seq from children with SLE (cSLE) has demonstrated that NK cells, with up-regulated cytotoxicity-encoding genes, were expanded in cSLE patients [29]. Discrepancy in the results may be explained by differences in ages, levels of disease activities and treatment regimen, as SLE is a heterogeneous autoimmune disease. Our research suggests that altered NK cell subsets and impaired function might impede their ability to eradicate activated pathogenic CD4^+^ T cells.

On the other hand, SLE patients are susceptible to infections which have also been shown to play a role in the development and exacerbation of SLE [2, 30, 31]. NK cell deficiency and its blunted cytotoxicity function may be an important risk factor for infections in SLE patients, especially the opportunistic infections.

Novel drugs that target NK cells are currently being explored in cancers and autoimmune diseases [32]. Atopic dermatitis (AD) patients show deficiency in circulating NK cells and administration of NK cell-boosting IL-15 superagonist leads to clinical amelioration in AD mice [33]. Daclizumab (targeting IL-2Ralpha chain) therapy may relieve the symptoms of multiple sclerosis patients, which is associated with expansion of CD56^bright^ NK cells and a gradual decline in circulating CD4^+^ T cells [34]. Restoring NK cell activity may contribute to the elimination of plasma cells from SLE patients, and elotuzumab (targeting SLAMF7) and daratumumab (targeting CD38) may contribute to the elimination of antibody producing cells in vitro [35]. Recent studies have verified that low-dose IL-2 treatment was effective in SLE patients and could increase NKG2D^+^ NK cells [36]. Taken together, it is justifiable to suppose that restoration of NK cell activity (e.g. through low-dose IL-2) could potentially help regain immune balance and minimize both systemic autoimmunity and the risk of infection in SLE patients.

Oxidative stress characterizes the lupus immune system and promotes its pathogenesis at multiple levels [37, 38]. Treatment with MitoTempo, an inhibitor of mtROS production, delays disease progression in lupus mice [37]. And treatment with the ROS scavenger N-acetyl-cysteine (NAC) relieves disease activity of SLE patients in a randomized, double-blind, placebo-controlled trial study [38]. ROS facilitates metastasis of melanoma cells by down-regulating functions of NK cells, and ROS inhibition may restore NK cell-mediated clearance of these malignant cells [39]. Our study confirmed that the elevated levels of ROS and mtROS in CD56^dim^CD57^+^NK cells from lupus patients were associated with increased apoptosis and decreased function of NK cells. A previous research has demonstrated that CD56^dim^ NK cells, but not CD56^bright^ NK cells, are likely to succumb to apoptosis when undergoing oxidative stress [40]. Consistent with this view, CD56^bright^ NK cells did not change significantly in our study. We further indicated that, in the CD56^dim^ NK cell population, CD57^+^ cells suffered from higher oxidative stress and more cell apoptosis, which may explain the selective reduction of CD56^dim^CD57^+^ NK cells seen in lupus patients.

We recognized that there were several limitations in our study. The total number of patients was somewhat small and larger prospective cohorts are required to perform further analyses. We described the contribution of ROS to the function of NK cell subsets using in vitro experiments, further investigations conducted in vivo will be more valuable. And the molecular pathways involved in the cross-talk between CD4^+^ T cells and NK cells need further research.

## Conclusion

In summary, we demonstrated decreased number and impaired in vitro function of CD56^dim^CD57^+^ NK cells in SLE patients. This NK cell subset was negatively correlated with the severity of SLE patients. The selective reduction may due to preferential apoptosis of CD56^dim^CD57^+^ NK cells under increased oxidative stress in SLE patients. More importantly, CD56^dim^CD57^+^ NK cells exhibited stronger cytotoxicity in response to activated CD4^+^ T cells. Altered NK cell subsets and impaired function might impede their ability to eradicate activated pathogenic CD4^+^ T cells which are key cells that cause lupus disease.

## Supplementary Information

**Additional file 1: Supplementary Table 1. **Antibody list. **Supplementary Table 2. **Baseline characteristics of patients and healthy controls in this study. **Supplementary Table 3. **Hallmark gene sets((Lupus vs Control)*. **Supplementary Table 4. **Hallmark gene sets((CD57^+^ vs CD57^-^)*. **Supplementary Figure 1. **The NK cell subset gating strategies. **Supplementary Figure 2. **Supplementary analysis of clinical data and peripheral NK count in SLE and RA patients. **Supplementary Figure 3. **The correlation of CD56^dim^CD57^+^ NK cell percentage with SLEDAI (n=37). **Supplementary Figure 4.** Apoptosis and ROS levels of sorted NK cells upon exposure to H_2_O_2_. **Supplementary Figure 5. **Cytokine expression of CD56^dim^CD57^+^ NK cells in SLE patients and HCs. **Supplementary Figure 6. **No cytotoxicity of resting CD4^+^ T cells by NK cells(n=3). **Supplementary Figure 7. ** PD-1 expression of NK cell subsets in SLE patients (n=8) and HCs (n=8).

## Data Availability

The data used and/or analyzed in the current study are available from the corresponding author on reasonable request.

## Abbreviations

SLE: Systemic lupus erythematosus
RA: Rheumatoid arthritis
ACR: American College of Rheumatology
HC: Healthy control
PLT: Blood platelet
C3: Serum concentrations of complement C3
C4: Serum concentrations of complement C4
anti-dsDNA antibodies: Anti-double-stranded-DNA antibodies
ANA: Antinuclear antibodies
IgG: Immunoglobulin G
IgM: Immunoglobulin M
IgA: Immunoglobulin A
SLEDAI: Systemic Lupus Erythematosus Disease Activity Index
DAS28: 28-Joint disease activity score
LN: Lupus nephritis
PBMCs: Peripheral blood mononuclear cells
PMA: Phorbol 12-myristate 13-acetate
7-AAD: 7-Amino-actinomycin D
ROS: Reactive oxygen species
mtROS: Mitochondrial reactive oxygen species
DCFH-DA: 2',7'-Dichlorodihydrofluorescein diacetate
MFI: Mean fluorescence intensity
MACS: Magnet associated cell sorting
FACS: Fluorescence activated cell sorting
FBS: Foetal Bovine Serum
CFSE: Carboxyfluorescein diacetate succinimidyl ester
FVD: Fixable Viability Dyes
H_2_O_2_: Hydrogen peroxide
t-SNE: T-distributed stochastic neighbour embedding
GSEA: Gene Set Enrichment Analysis
GEO: Gene Expression Omnibus
NES: Normalized enrichment score
MSigDB: Molecular Signature v7.2 Database
IQR: Interquartile range
ICOS: Inducible T cell costimulator
PD-1: Programmed death 1
