# Supplementary material for: Increased oxidative stress contributes to impaired peripheral CD56dimCD57+ NK cells from patients with systemic lupus erythematosus
Source: Arthritis Res Ther. 2022 Feb 16;24:48. doi: 10.1186/s13075-022-02731-y (PMC8848960; doi:10.1186/s13075-022-02731-y)
Supplement: Supplementary file 1 — Additional file 1: Supplementary Table 1. Antibody list. Supplementary Table 2. Baseline characteristics of patients and healthy controls in this study. Supplementary Table 3. Hallmark gene sets((Lupus vs Control)*. Supplementary Table 4. Hallmark gene sets((CD57+ vs CD57-)*. Supplementary Figure 1. The NK cell subset gating strategies. Supplementary Figure 2. Supplementary analysis of clinical data and peripheral NK count in SLE and RA patients. Supplementary Figure 3. The correlation of CD56dimCD57+ NK cell percentage with SLEDAI (n=37). Supplementary Figure 4. Apoptosis and ROS levels of sorted NK cells upon exposure to H2O2. Supplementary Figure 5. Cytokine expression of CD56dimCD57+ NK cells in SLE patients and HCs. Supplementary Figure 6. No cytotoxicity of resting CD4+ T cells by NK cells(n=3). Supplementary Figure 7. PD-1 expression of NK cell subsets in SLE patients (n=8) and HCs (n=8). [file 13075_2022_2731_MOESM1_ESM.doc]

**Appendices**

**Table of Contents**

**1.Supplementary Table1. Antibody list** **-------------------------------------------------------------------------------2**

**2.Supplementary Table2. Baseline characteristics of patients and healthy controls in this study--------------------------------------------------------------------------------------------------------------------------3**

**3.Supplementary Table3. Hallmark gene sets(Lupus vs Control)-----------------------------------------------4**

**4.Supplementary Table4. Hallmark gene sets(CD57^+^ vs CD57^-^)-------------------------------------------------5**

**5. Supplementary Figure1. The NK cell subset gating strategies ---------------------------------------------6**

**6. Supplementary Figure2. Supplementary analysis of clinical data and peripheral NK count in SLE patients ---------------------------------------------------------------------------------------------------------------------7**

**7. Supplementary Figure3. The correlation of CD56^dim^CD57^+^ NK cell percentage with SLEDAI-------8**

**8. Supplementary Figure4. Apoptosis and ROS levels of sorted NK cells upon exposure to H_2_O_2_--------------------------------------------------------------------------------------------------------------------------9**

**9. Supplementary Figure5. Cytokine expression of CD56^dim^CD57^+^NK cells**  **------------------------------10**

**10. Supplementary Figure6. No cytotoxicity of resting CD4^+^ T cells by NK cells ------------------------11**

**11.Supplementary Figure7. PD-1 expression of NK cell subsets in SLE patients and HCs---------------12**

**Supplementary Table 1. Antibody list**

| REAGENT | SOURCE | Clone |
| --- | --- | --- |
| Anti-human-CD45-V500 | BD Biosciences | HI30 |
| Anti-human-CD3-percp | Biolegend | HIT3A |
| Anti-human-CD56-BV421 | Biolegend | HCD56 |
| Anti-human-CD56-PE | Biolegend | HCD56 |
| Anti-human-CD3-Alexa Flauor 488 | Biolegend | HIT3A |
| Anti-human-CD3-APC | Biolegend | HIT3A |
| Anti-human-CD56-APC | Biolegend | 5.1H11 |
| Anti-human-CD56-percp | Biolegend | 5.1H11 |
| Anti-human-CD57-PE-Cy7 | Biolegend | HNK-1 |
| Anti-human-CD38-FITC | Biolegend | HIT2 |
| Anti-human-CD20-PE | BD Biosciences | 2H7 |
| Anti-human-CD19-APC | Biolegend | HIB19 |
| Anti-human-CD27-APC/Cyanine7 | Biolegend | O323 |
| Anti-human-CD16-FITC | Biolegend | 3J8 |
| Anti-human-CD107a-PE | Biolegend | H4A3 |
| Anti-human-PD-1-FITC | Biolegend | EH12.2H7 |
| Anti-human-ICOS-PE | eBioscience | ISA-3 |
| Anti-human-perforin-FITC | Biolegend | dG9 |
| Anti-human-Granzyme B-PE | Biolegend | QA16A02 |
| Anti-human-NKG2D-APC | Biolegend | 1D11 |
| Anti-human-NKG2A-FITC | miltenyi | REA110 |
| Anti-human-IFNγ-APC | eBioscience | 4S.B3 |
| Anti-human-IL-10-APC | Biolegend | JES3-9D7 |
| Anti-human-Cleaved-caspase3(Asp175)-Alexa Flauor 647 | CST | D3E9 |
| Anti-human-Cleaved-caspase9(Asp315)-PE | CST | D8I9E |
| Anti-human-Foxp3-APC | eBioscience | PCH101 |

**Supplementary Table 2. Baseline characteristics of patients and healthy controls in this study.**

| Characteristics | SLE(n=156) | RA(n= 22) | HC(n=30) | P |
| --- | --- | --- | --- | --- |
| Age, year | 33 (26-47) | 37 (32-47) | 32 (27-38) | 0.146 |
| Gender(Female/Male) | 141/15 | 20/2 | 27/3 | 0.994 |
| ANA ,n (%) | 156 (100%) | - | - | **-** |
| C3, g/L | 0.45±0.22 | - | - | **-** |
| C4, g/L | 0.095±0.066 | - | - | **-** |
| Anti-dsDNA antibody, IU/mL | 217 (43-700) | - | - | **-** |
| Hemocytopenia,n (%) | 112 (71.8%) |  |  |  |
| New rashes ,n (%) | 96 (61.5%) | - | - | **-** |
| Arthritis,n (%) | 67 (42.9%) | - | - | **-** |
| CNS involvement,n (%) | 9 (5.77%) | - | - | **-** |
| Lupus nephritis ,n (%) | 74 (47.4%) | - | - | **-** |
| DAS28 | - | 4.95±1.55 | - | **-** |
| SLEDAI | 11.7±5.7 | - | - | **-** |

For a continuous variable, median (IQR), or mean±SD. For a categorical variable, count (percentage).

SLE: systemic lupus erythematosus; RA: rheumatoid arthritis; HC: healthy control; ANA: antinuclear antibodies; C3: complement 3; anti-dsDNA antibodies: anti-double-stranded-DNA antibodies; CNS: central nervous system; DAS28: 28-joint disease activity score; SLEDAI: Systemic Lupus Erythematosus Disease Activity Index 2000.

**Supplementary Table 3. Hallmark gene sets((Lupus vs Control)***

| Description | NES | p value | FDR |
| --- | --- | --- | --- |
| HALLMARK_INFLAMMATORY_RESPONSE | 1.791340646 | 0.000155545 | 0.000451773 |
| HALLMARK_COMPLEMENT | 1.835984374 | 0.000155569 | 0.000451773 |
| HALLMARK_INTERFERON_GAMMA_RESPONSE | 2.051223492 | 0.000155618 | 0.000451773 |
| HALLMARK_TNFA_SIGNALING_VIA_NFKB | 1.861956539 | 0.000156055 | 0.000451773 |
| HALLMARK_HYPOXIA | 1.727491651 | 0.000156348 | 0.000451773 |
| HALLMARK_HEME_METABOLISM | 1.819620146 | 0.00015647 | 0.000451773 |
| HALLMARK_IL6_JAK_STAT3_SIGNALING | 2.045528527 | 0.000170155 | 0.000451773 |
| HALLMARK_INTERFERON_ALPHA_RESPONSE | 2.120465615 | 0.000171674 | 0.000451773 |
| HALLMARK_DNA_REPAIR | -1.860396452 | 0.00026448 | 0.000618667 |
| HALLMARK_XENOBIOTIC_METABOLISM | 1.647093711 | 0.001092385 | 0.002299757 |
| HALLMARK_COAGULATION | 1.674427483 | 0.001454428 | 0.002783594 |
| HALLMARK_ALLOGRAFT_REJECTION | -1.530500093 | 0.001944444 | 0.003411306 |
| HALLMARK_KRAS_SIGNALING_UP | 1.553233742 | 0.003434817 | 0.005562456 |
| HALLMARK_G2M_CHECKPOINT | 1.542336698 | 0.004217432 | 0.00614227 |
| HALLMARK_MITOTIC_SPINDLE | 1.527246102 | 0.004376368 | 0.00614227 |
| HALLMARK_APICAL_SURFACE | -1.685100125 | 0.00654037 | 0.008156246 |
| HALLMARK_ANGIOGENESIS | 1.678028939 | 0.006586169 | 0.008156246 |
| HALLMARK_ESTROGEN_RESPONSE_LATE | 1.477377153 | 0.007802747 | 0.009126019 |
| HALLMARK_GLYCOLYSIS | 1.440859332 | 0.010278773 | 0.011389222 |
| HALLMARK_E2F_TARGETS | 1.43750978 | 0.012293806 | 0.012940849 |
| HALLMARK_APICAL_JUNCTION | 1.403605338 | 0.016859195 | 0.016901448 |
| HALLMARK_PROTEIN_SECRETION | 1.460646468 | 0.023576962 | 0.022548702 |
| HALLMARK_APOPTOSIS | 1.389509186 | 0.024634456 | 0.022548702 |
| HALLMARK_IL2_STAT5_SIGNALING | 1.367850585 | 0.026766262 | 0.023479177 |
| HALLMARK_REACTIVE_OXYGEN_SPECIES_PATHWAY | 1.528012395 | 0.029019466 | 0.02358904 |
| HALLMARK_UV_RESPONSE_UP | 1.382281707 | 0.029132464 | 0.02358904 |
| HALLMARK_MTORC1_SIGNALING | 1.330392415 | 0.036846214 | 0.028729991 |
| HALLMARK_UV_RESPONSE_DN | 1.350494041 | 0.040077569 | 0.030133511 |
| HALLMARK_PEROXISOME | 1.382753747 | 0.044385116 | 0.031942678 |
| HALLMARK_P53_PATHWAY | 1.316530593 | 0.045518316 | 0.031942678 |

*Note. Since there was no appropriate data of human NK cells, we adopted NK cell data from mouse SLE. Though the marker of mouse NK cells is different from that of human NK cells, but they have similar functions and corresponding subpopulations^[1]^.

[1] Hayakawa Y, et al. Functional subsets of mouse natural killer cells. Immunol Rev. 2006 Dec;214:47-55. doi: 10.1111/j.1600-065X.2006.00454.x.

**Supplementary Table 4. Hallmark gene sets((CD57^+^ vs CD57^-^)***

| Description | NES | p value | FDR |
| --- | --- | --- | --- |
| HALLMARK_OXIDATIVE_PHOSPHORYLATION | 1.452493566 | 0.007518797 | 0.165260078 |
| HALLMARK_PROTEIN_SECRETION | 1.54839664 | 0.007640879 | 0.165260078 |
| HALLMARK_INFLAMMATORY_RESPONSE | -1.395885328 | 0.010704346 | 0.165260078 |
| HALLMARK_REACTIVE_OXYGEN_SPECIES_PATHWAY | 1.572815787 | 0.017639077 | 0.204241948 |
| HALLMARK_E2F_TARGETS | 1.342046737 | 0.022667666 | 0.209974167 |

*Note. The two subsets in GSE23695 are CD3^-^CD56^dim^CD16^+^CD57^+^ and CD3^-^CD56^dim^CD16^+^CD57^-^ NK cells. In peripheral blood of healthy people, CD56^dim^ NK cells express high levels of CD16. So we use that data for our study comparing CD56^dim^CD57^+^ and CD56^dim^CD57^-^ NK cells.


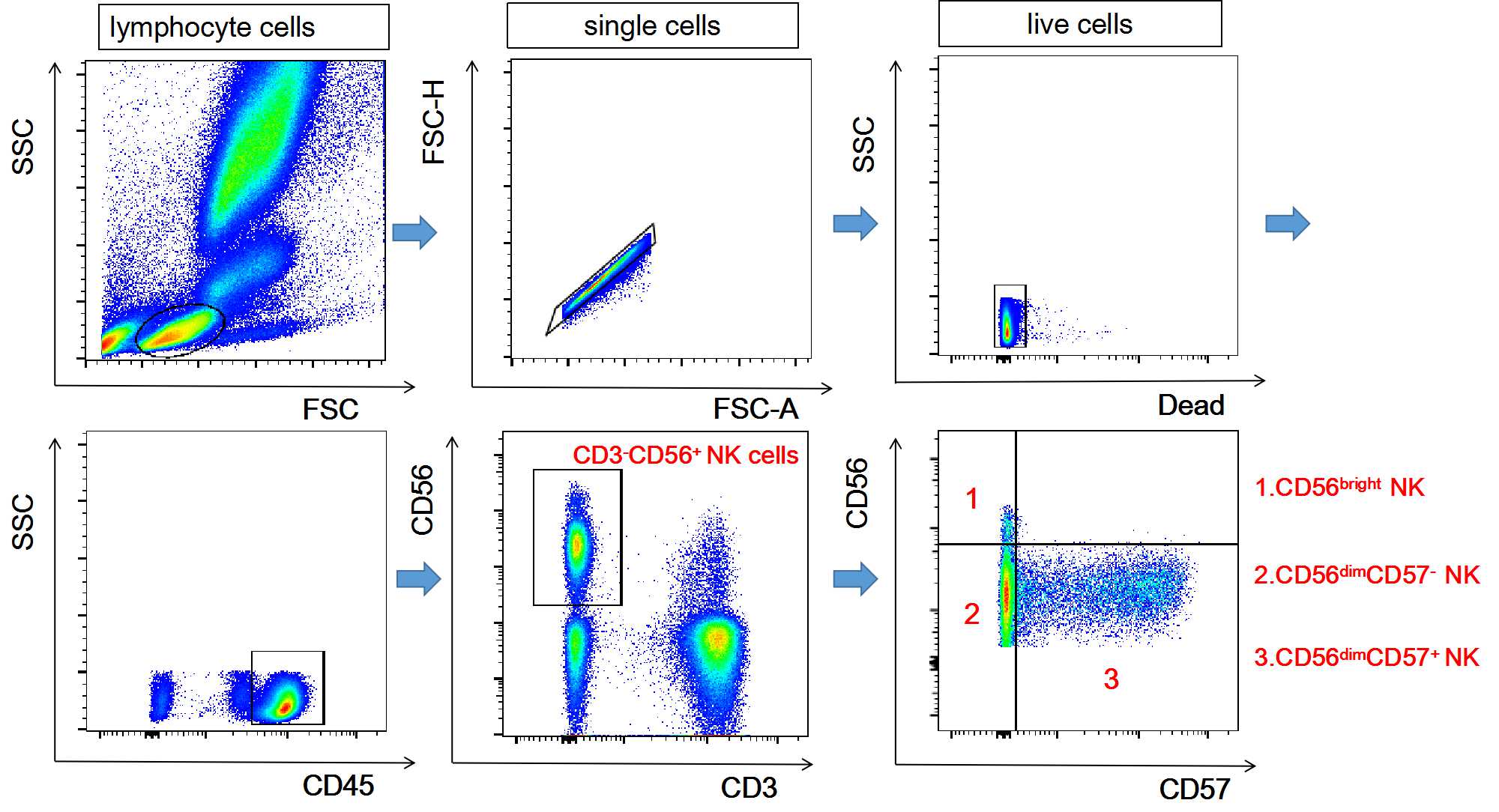


**Supplementary Figure1.** The NK cell subset gating strategies.

Lymphocytes were gated on forward scatter(FSC) and side scatter(SSC) characteristics, then single cells on forward scatter height(FSC-H) versus forward scatter area(FSC-A), followed by a gate on live, CD45^+^CD3^–^CD56^+^ NK cells. NK cell subsets(CD56^bright^ NK cells, CD56^dim^CD57^-^ NK cells, and CD56^dim^CD57^+^ NK cells) were defined according to CD56 and CD57 expression.


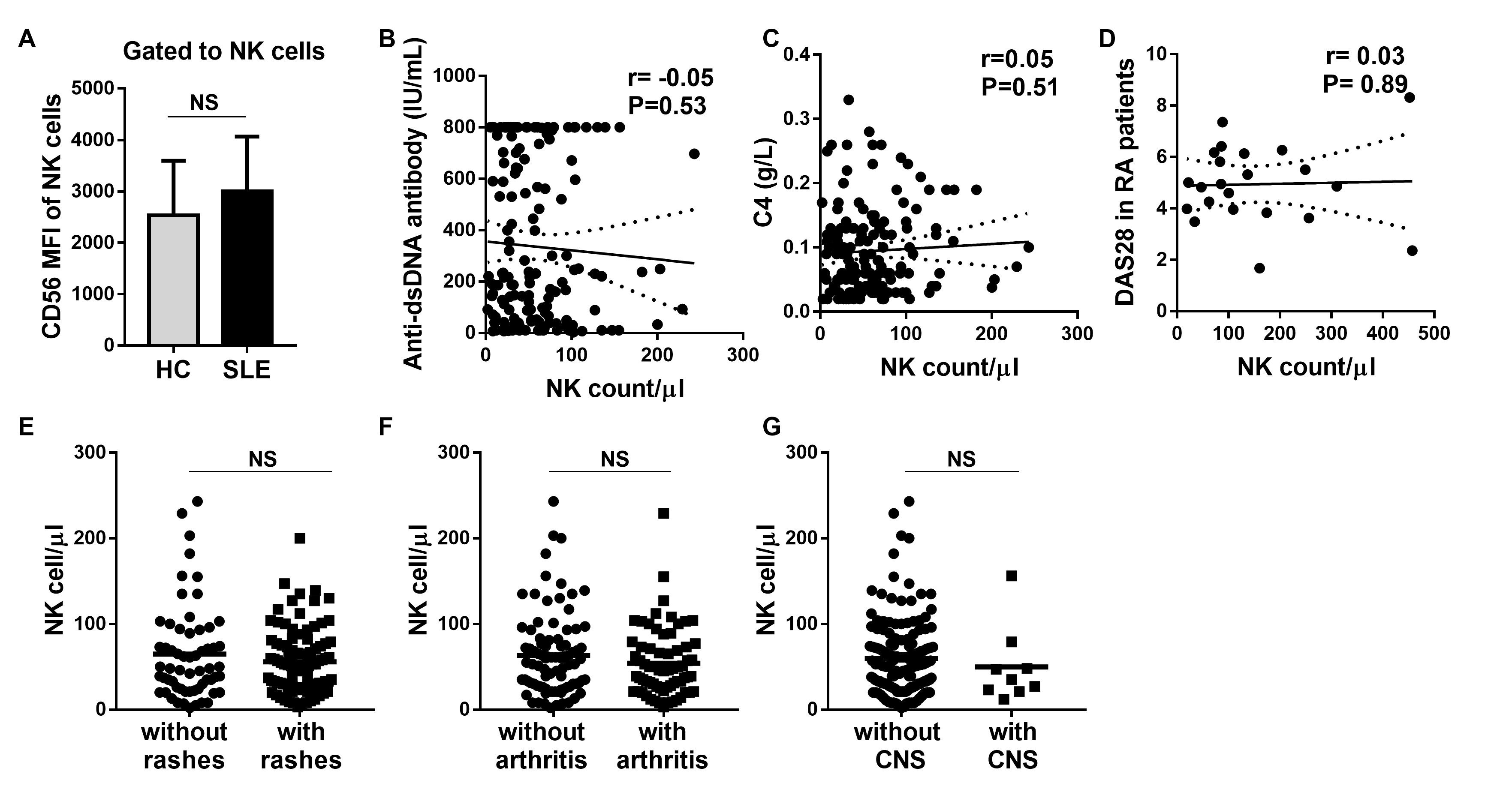


Supplementary Figure2. Supplementary analysis of clinical data and peripheral NK count in SLE and RA patients.

1. Comparison of CD56 MFI of peripheral NK cells in SLE patients and HCs.
2. Correlation of the NK count with the level of anti-dsDNA antibody in SLE patients.
3. Correlation of the NK count with the level of C4 in SLE patients.
4. Correlation of the NK count with DAS28 in RA patients.
5. Comparison of the absolute number of peripheral NK cells in SLE patients with (n=96) and without new rashes (n=60).
6. Comparison of the absolute number of peripheral NK cells in SLE patients with (n=67) and without arthritis (n=89).
7. Comparison of the absolute number of peripheral NK cells in SLE patients with (n=9) and without CNS involvement (n=147).

DAS28: 28-joint disease activity score; CNS: central nervous system


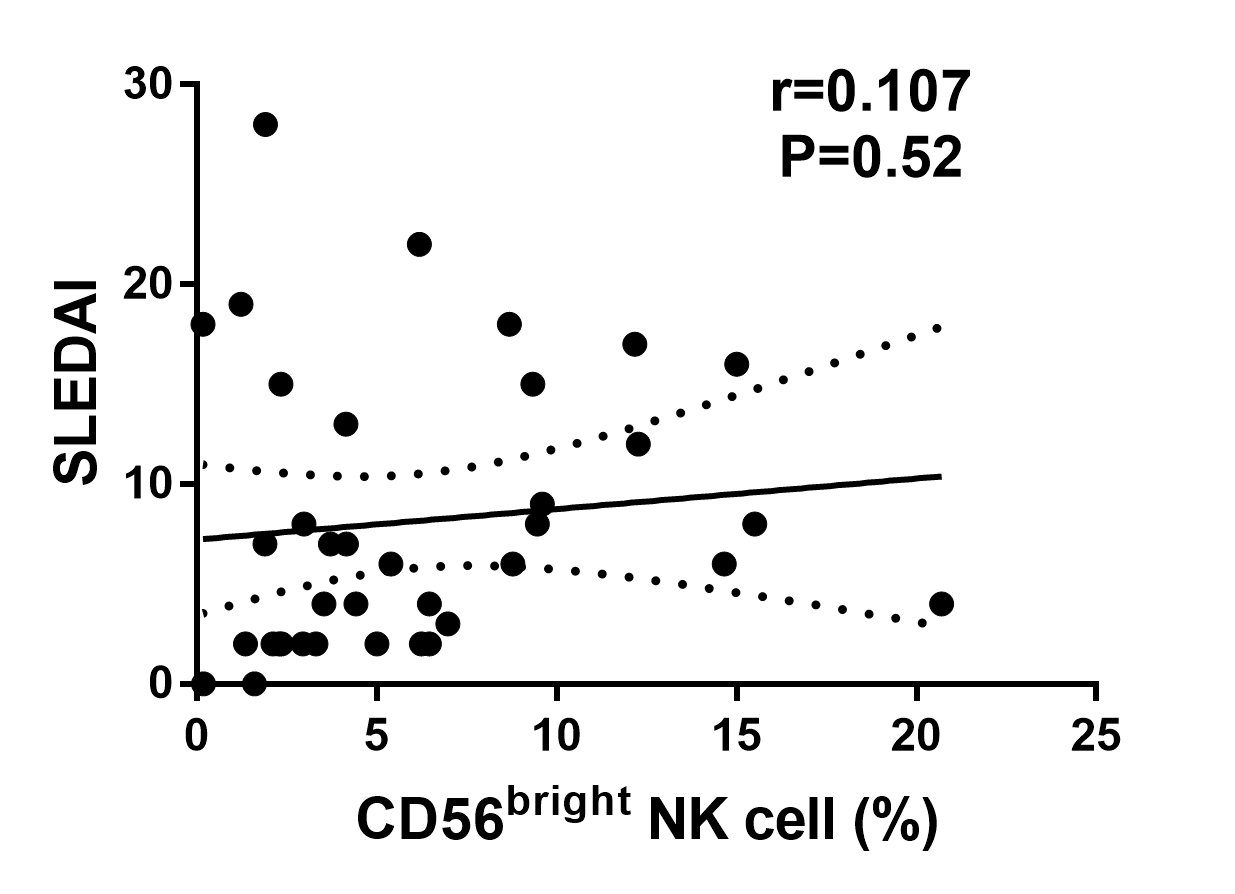


Supplementary Figure3. The correlation of CD56^dim^CD57^+^ NK cell percentage with SLEDAI (n=37).


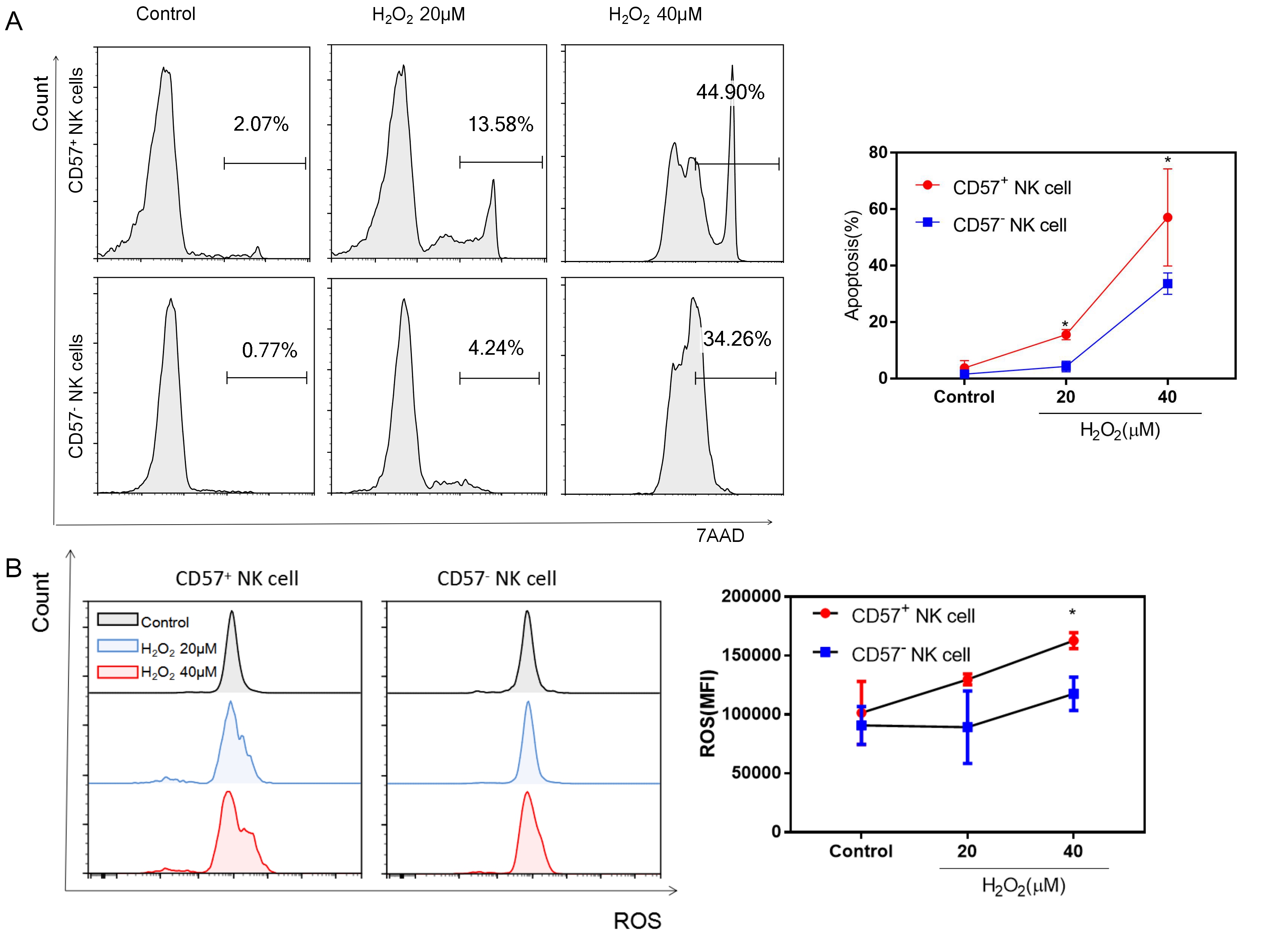


Supplementary Figure4. Apoptosis and ROS levels of sorted NK cells upon exposure to H_2_O_2_.

1. Comparison of apoptosis level of CD57^+^ NK cell subset and CD57^-^ NK cell subset from sorted NK cells upon exposure to H_2_O_2_ for 24 hour culture at concentrations of 20µM and 40µM, respectively (n=3).
2. Comparison of ROS level of CD57^+^ NK cell subset and CD57^-^ NK cell subset from sorted NK cells upon exposure to H_2_O_2_ for 24 hour culture at concentrations of 20µM and 40µM, respectively (n=3).

(* P< 0.05).


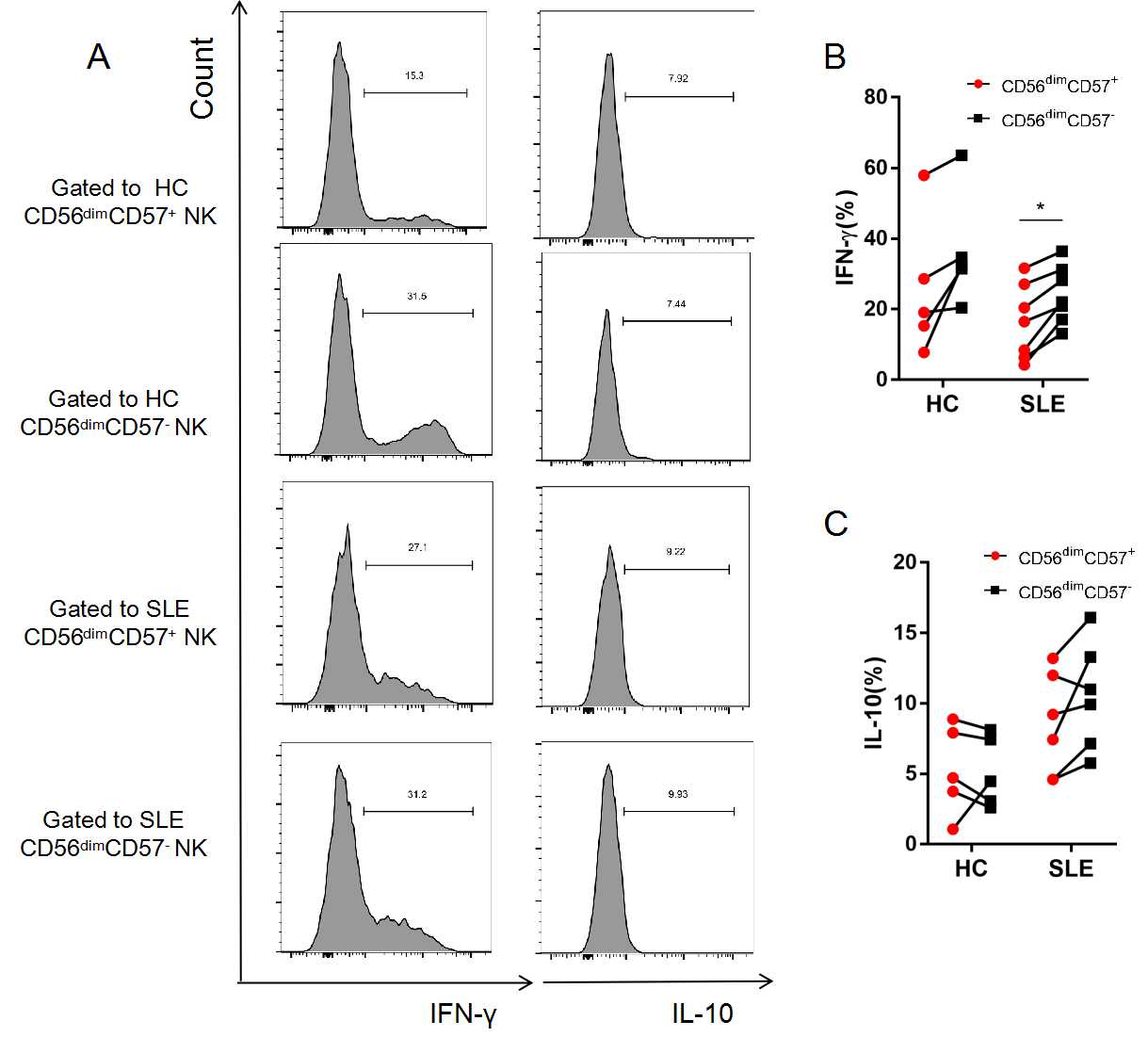


**Supplementary Figure 5.** Cytokine expression of CD56^dim^CD57^+^ NK cells in SLE patients and HCs.

1. Representative flow cytometric plot for expression of IFN-γ and IL-10 on NK cell subsets (CD56^dim^CD57^+^ NK cell subset and CD56^dim^CD57^-^ NK cell subset) in HC and SLE patient.
2. Comparison of IFN-γ expression of on NK cell subsets in SLE patients (n = 7) and HCs (n = 5).
3. Comparison of IL-10 expression of on NK cell subsets in SLE patients (n = 6) and HCs (n = 5).

(* P< 0.05).


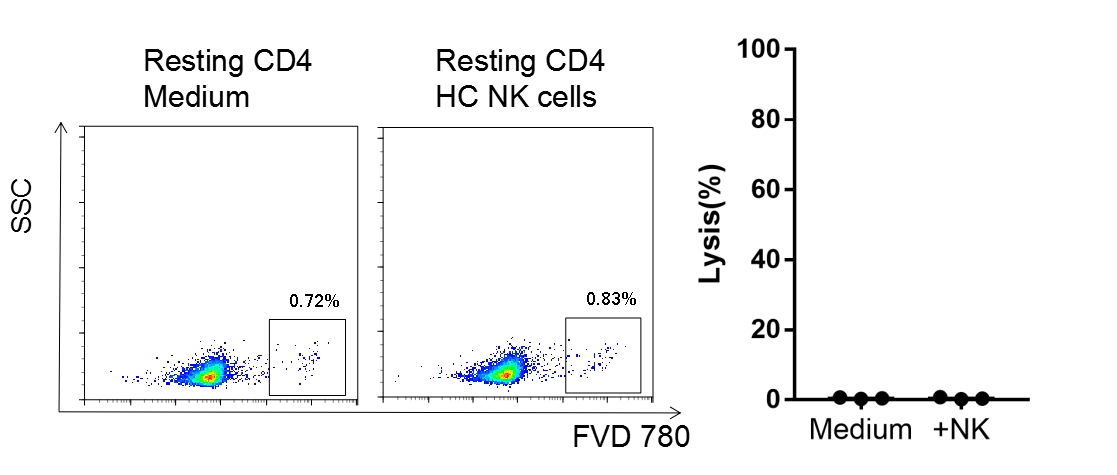


**Supplementary Figure6.** No cytotoxicity of resting CD4^+^ T cells by NK cells(n=3).


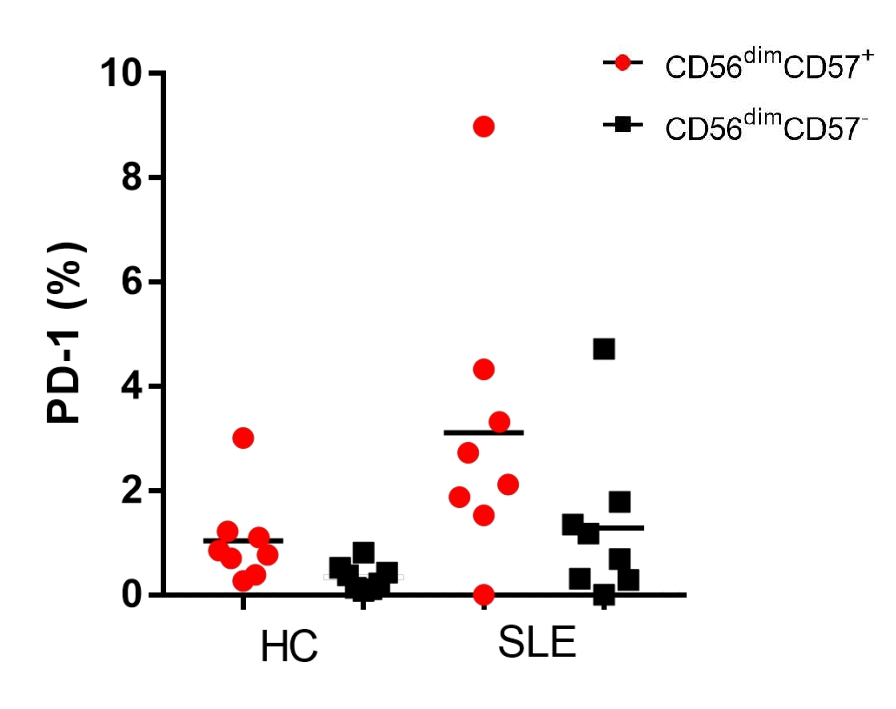


Supplementary Figure7. PD-1 expression of NK cell subsets in SLE patients (n=8) and HCs (n=8).
